# Periodic and Spatial Spreading of Alkanes and *Alcanivorax* Bacteria in Deep Waters of the Mariana Trench

**DOI:** 10.1128/AEM.02089-18

**Published:** 2019-01-23

**Authors:** Wen-Li Li, Jiao-Mei Huang, Pei-Wei Zhang, Guo-Jie Cui, Zhan-Fei Wei, Yu-Zhi Wu, Zhao-Ming Gao, Zhuang Han, Yong Wang

**Affiliations:** a Institute of Deep-Sea Science and Engineering, Chinese Academy of Sciences, Sanya, Hainan, China; b University of Chinese Academy of Sciences, Beijing, China; North Carolina State University

**Keywords:** *Alcanivorax*, alkanes, deep sea, subduction zone

## Abstract

In subduction zones, serpentinization and biological processes may release alkanes to the deep waters, which would probably result in the rapid spread of *Alcanivorax*. However, the timing and area of the alkane distribution and associated enrichment of alkane-degrading microbes in the dark world of the deep ocean have not been explored. In this study, we report the richness (up to 17.8%) of alkane-degrading bacteria, represented by Alcanivorax jadensis, in deep water samples obtained at 3,000 to 6,000 m in the Mariana Trench in two cruises. The relative abundance of A. jadensis correlated with copy numbers of functional *almA* and *alkB* genes, which are involved in alkane degradation. In these water samples, we detected a high flux of alkanes, which probably resulted in the prevalence of A. jadensis in the deep waters. Contigs of A. jadensis were binned from the metagenomes for examination of alkane degradation pathways and deep sea-specific pathways, which revealed a lack of nitrate and nitrite dissimilatory reduction in our A. jadensis strains. Comparing the results for the two cruises conducted close to each other, we suggest periodic release of alkanes that may spread widely but periodically in the trench. Distribution of alkane-degrading bacteria in the world’s oceans suggests the periodic and remarkable contributions of *Alcanivorax* to the deep sea organic carbon and nitrogen sources.

**IMPORTANCE** In the oligotrophic environment of the Mariana Trench, alkanes as carbohydrates are important for the ecosystem, but their spatial and periodic spreading in deep waters has never been reported. Alkane-degrading bacteria such as *Alcanivorax* spp. are biological signals of the alkane distribution. In the present study, *Alcanivorax* was abundant in some waters, at depths of up to 6,000 m, in the Mariana Trench. Genomic, transcriptomic, and chemical analyses provide evidence for the presence and activities of Alcanivorax jadensis in deep sea zones. The periodic spreading of alkanes, probably from the subductive plates, might have fundamentally modified the local microbial communities, as well as perhaps the deep sea microenvironment.

## Abstract (precis)

In the oligotrophic environment of the Mariana Trench, alkanes as carbohydrates are important for the ecosystem, but their spatial and periodic spreading in deep waters has never been reported. Alkane-degrading bacteria such as *Alcanivorax* spp. are biological signals of the alkane distribution. In the present study, *Alcanivorax* was abundant in some waters, at depths of up to 6,000 m, in the Mariana Trench. Genomic, transcriptomic, and chemical analyses provide evidence for the presence and activities of Alcanivorax jadensis in deep sea zones. The periodic spreading of alkanes, probably from the subductive plates, might have fundamentally modified the local microbial communities, as well as perhaps the deep sea microenvironment.

## INTRODUCTION

Away from the eutrophic zone, deep sea organisms thriving in lower mesopelagic to hadal realms confront a nutrient-deprived environment (1). Given the fact that euphotic export and chemolithoautotrophic biosynthesis in the water column can contribute organic carbon to these deep layers (1–3), there is still discrepancy in the carbon budget in the dark ocean. It is controversial how much the puzzle will be resolved by extra organic carbon input from subsurface marine sediments and even the crust of the Earth. Movement of tectonic plates generates geothermal gases represented by methane, which is accumulating in marginal slopes and being released from deep sea vents, mud volcanoes, and pockmarks (4). There are also considerable amounts of CH_4_ and CO_2_ in the hydrothermal solution seeping out from deep ocean ridges and rifts (5, 6). The inorganic carbon is assimilated for microbial autotrophic growth. Therefore, tectonic activities beneath the seafloor contribute dramatically to the carbon budgets (7, 8). The role of subduction zones between convergent plates has not been fully evaluated, although special microbial lineages resembling those in early life were discovered in the 10-km-deep mantle, mud volcano, and cold seep in serpentinized sites of the Izu-Bonin-Mariana forearc and Lost City hydrothermal field (9–11). In addition to high concentrations of hydrogen and methane in serpentinized seepage fluids, there were also *n*-alkanes such as butane and propane (12). In experiments under simulation conditions, the serpentinization process may yield alkanes up to C_27_, which has been detected in fluid samples from the Rainbow hydrothermal field (13). However, alkanes were not detected in most of the previous studies (12), and how far alkanes can spread in deep sea waters was not investigated. Therefore, the contribution of alkanes to the deep ocean carbon budget is probably underestimated, as subduction zones are widely distributed along the convergent plates in the oceans.

Aside from thermogenic alkanes, decomposition of fatty acids from bacteria and algae also produces alkanes, particularly in anoxic sediments (14). Alkanes may be anaerobically degraded by nitrate- or sulfate-reducing bacteria but are more efficiently oxidized by aerobic microorganisms in open oceans (15). A large number of studies have shown the explosive prevalence of alkane-degrading bacteria in waters after oil pollution (16, 17). For example, Alcanivorax borkumensis SK2 and Alcanivorax jadensis T9 are able to utilize *n*-alkanes ranging from C_5_ to C_32_, depending on the *alk* and *alm* gene families, which are specialized for intermediate- and high-molecular-weight alkanes, respectively (18, 19). Their presence is a microbial signal for leakage of alkanes in waters. For deep sea zones, the alkane degradation mediated by microbes has rarely been reported (20). There is no direct evidence for the association between microbial degradation of alkanes and the serpentinization process. Probably alkanes are anaerobically degraded in sediments or injected into water columns. However, *Alcanivorax* species and other alkane-degrading bacteria were not detected in serpentinization sediments, fluids, and adjacent waters. Complete consumption of alkanes by microbes is occasionally limited by insufficient nitrogen and phosphate supplies (21). Depletion of oxygen may also hinder the removal of heavy alkanes from oil-polluted areas. The slow degradation permits long-distance spread of alkanes to form a plume after strong seepage of thermogenic or biogenic alkanes. The hypothesis of the formation of alkane plumes in dark oceans has not been substantiated to date.

The Mariana Trench was formed by subduction of the western North Pacific plate under the Philippine plate, which resulted in the deepest site on Earth, Challenger Deep. Recently, a cold seep was discovered on the forearc slope of the trench (10). The ecosystem was likely fueled by serpentinized fluids of high pH (10), which is supported by the evidence of serpentinization in the drilling core obtained in the nearby forearc (11). Alkane-degrading microbes have not been examined in the vents and approximate bottom waters. Therefore, the potential influence of alkanes produced by serpentinization was not evaluated. In this study, we sampled the water columns in the Mariana Trench, aiming to answer the questions regarding the spreading of alkanes in the subduction zone and the contribution of hydrocarbons to the ecosystem of deep waters. Alkanes up to C_32_ were detected in water samples at depths ranging from 1,000 m to 6,200 m. The high concentrations of alkanes remarkably correlated with the richness of A. jadensis, indicating the spreading of alkanes in the formation of plumes.

## RESULTS

### Microbial community structures.

**FIG 1:**
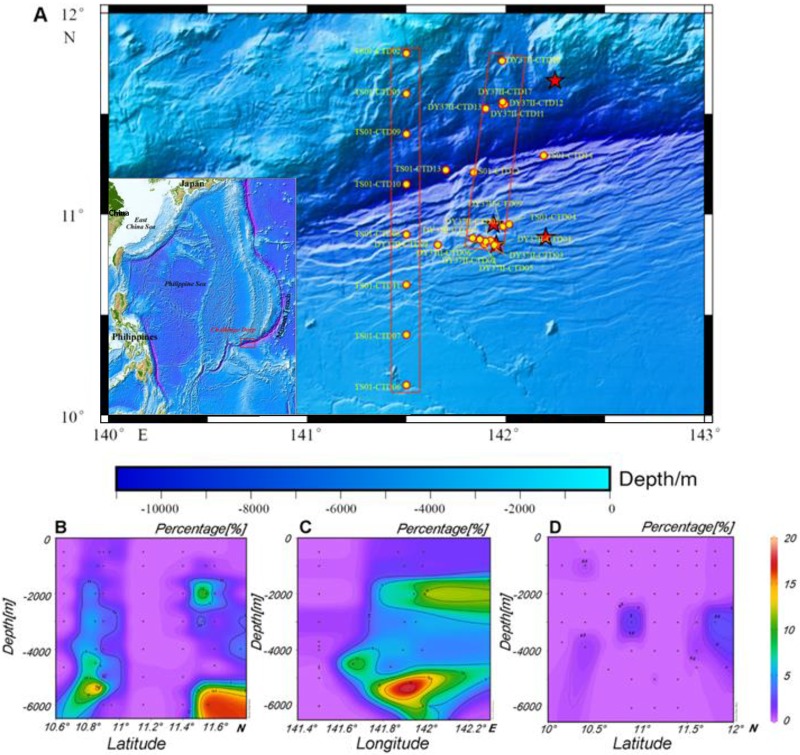
Collection of samples from the Mariana Trench. The water samples were collected with Niskin bottles (yellow dots) and a manned submersible (red stars) in two cruises (R/V *TS01* and *DY37II*) (A). Multiple sampling sites were selected to illustrate the vertical distribution of *Alcanivorax*. Both latitude (B) and longitude (C) distributions are depicted for the sites of the DY37II cruise, and the latitude distribution is shown for the samples collected in the TS01 cruise (D). The percentages of *Alcanivorax* in the mesopelagic and abyssal layers (500 to 6,000 m) were based on sequencing of the 16S rRNA gene amplicons (65). The inset map was created with Generic Mapping Tools (GMT) (66).

Along different water columns in the southern Mariana Trench, microbial communities were deciphered by sequencing 16S rRNA gene amplicons. The dominant bacterial groups in water samples were the *Proteobacteria* (mainly *Gammaproteobacteria* and *Deltaproteobacteria*), followed by the *Thaumarchaeota* and *Euryarchaeota* (see Fig. S1 in the supplemental material). The operational taxonomic units (OTUs) assigned to *Alcanivorax* were collected, to calculate their percentages at different depths (Table S1). The results showed that the relative abundance of *Alcanivorax* peaked at 17.8% in the 3,000-m water layer of the R/V *Dayang* no. 37-II (DY37II) conductivity, temperature, and depth 07 (CTD07) site (Fig. 1B). A prevalence of *Alcanivorax* (above 10%) was revealed in the water layers of 2,000 to 6,000 m at four sampling sites of the DY37II cruise (CTD01, CTD07, CTD12, and CTD17) (Fig. S1). In contrast, only a small percentage of OTUs for *Alcanivorax* was identified in the upper layers (<1,000 m). The distribution of *Alcanivorax* in bottom water (1 m above the bottom) and surface sediments was also examined. At four diving sites (5,460-m to 6,500-m depth) of the Jiaolong submersible (Fig. 1A), *Alcanivorax* was present only in the bottom waters and not in surface sediments (Fig. S2). The *Alcanivorax* in the bottom waters accounted for up to 5%, based on the 16S rRNA gene amplicon sequencing. For the samples from the R/V *Tansuo* no. 01 (TS01) cruise, the relative abundance of *Alcanivorax* was less than 1%. The TS01 samples were collected ∼2 months later than and ∼30 miles away from the DY37II samples, indicating spatial and periodic distribution of the *Alcanivorax* species in the Mariana deep waters.

### Genome binning of an *Alcanivorax* genome.

Metagenomes that were enriched in *Alcanivorax* were sequenced for DY37II CTD13 and DY37II CTD17. Genome binning obtained two genome bins for *Alcanivorax*, as indicated by the classification of the 16S rRNA genes (Fig. S3). The genome bin of *Alcanivorax* from DY37II CTD17 was 3.2 Mbp and comprised 88 contigs; that from DY37II CTD13 was 3.9 Mbp and comprised 159 contigs (Fig. S3). Assessment of the genome bins resulted in 95.5% and 85.6% completeness for those from DY37II CTD17 and DY37II CTD13, respectively, with low levels (<1.8%) of contaminants in the bins. The similarity of the two genome bins was >99% in reference to the average nucleotide identity (ANI), indicating that they belonged to the same species.

### Phylogenetic and correlation analyses.

**FIG 2:**
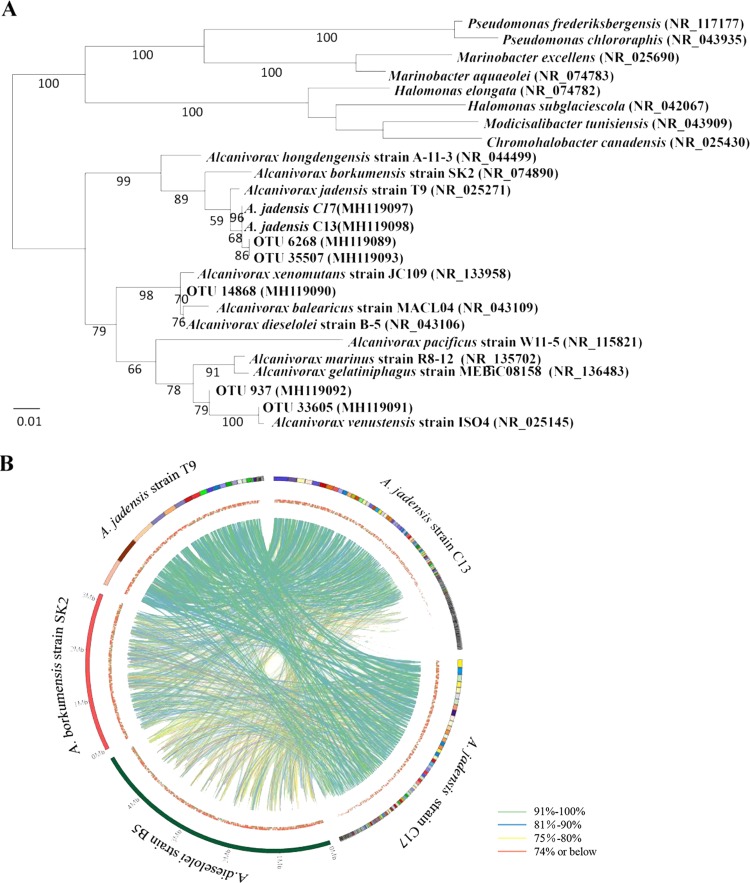
Phylogenetic relationships of major *Alcanivorax* species and plotting of genomic homologous regions. The genomes of A. jadensis strains C13 and C17 were binned from the metagenomes, and the OTUs for *Alcanivorax* were picked at 97% similarity. Reference 16S rRNA genes were pooled by BLASTn search of the NCBI databases, to reconstruct a phylogenetic tree based on a maximum-likelihood algorithm (A). Bootstrap values (displayed as percentages of 1,000 replications) are shown at the branches of the maximum-likelihood tree. The homologous regions between the five genomes are demonstrated by lines of different colors (B). The outer circle represents the contigs, and the inner circle depicts the predicted CDSs.

A 16S rRNA gene sequence was extracted from each of the genome bins. A phylogenetic tree based on the 16S rRNA sequences showed that the *Alcanivorax* species with the binned genomes were clustered with Alcanivorax jadensis strain T9 (Fig. 2A) (22). Since the 16S rRNA sequences in the Mariana Trench were almost identical to that of Alcanivorax jadensis strain T9 (99.9%), the binned *Alcanivorax* genomes were very likely derived from A. jadensis; their strain names were designated C13 and C17. Representative reads of 5 major OTUs classified as *Alcanivorax* were also examined for their phylogenetic positions. The 2 most abundant OTUs, i.e., OTUs 6268 and 35507, were almost identical to the 16S rRNA genes of A. jadensis strains C13 and C17. Two of the remaining 3 OTUs were close to Alcanivorax xenomutans and Alcanivorax venustensis. The two genome bins of A. jadensis strains C13 and C17 were compared with those of A. jadensis strain T9, Alcanivorax borkumensis strain SK2, and Alcanivorax dieselolei strain B5. Again, A. jadensis strains C13 and C17 were close relatives of A. jadensis strain T9, as indicated by the presence of a large number of homologous regions with high levels of similarity between their genomes (Fig. 2B).

### Metabolism specialization.

**FIG 3:**
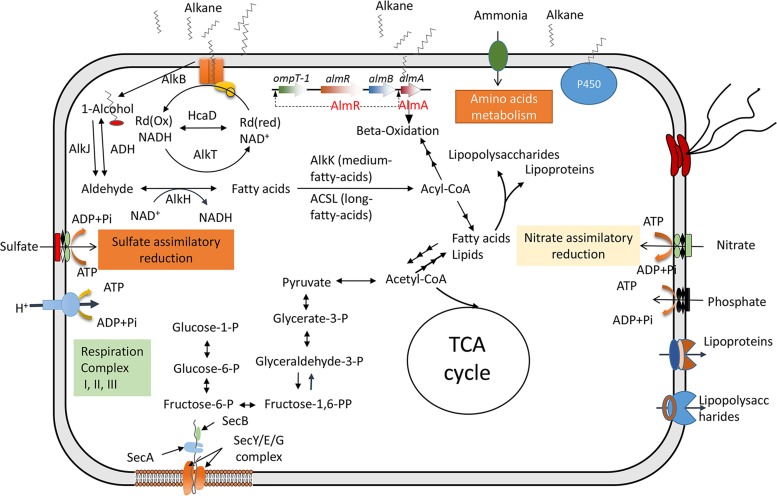
Schematic model of metabolism and cross-membrane transporters. The model was predicted based on the predicted genes in the genome bins of Alcanivorax jadensis strains C13 and C17. TCA, tricarboxylic acid.

The A. jadensis C13 and C17 genome bins contained three known alkane utilization pathways, mediated by cytochrome P450 and the Alk and Alm alkane monooxygenase systems (Fig. 3). There was a complete set of genes involved in lipid production and degradation in the A. jadensis C13 and C17 genome bins. Sulfate and nitrate might be imported for assimilatory reduction, as indicated by the identification of the related genes. We also compared the Kyoto Encyclopedia of Genes and Genomes (KEGG) annotations for five *Alcanivorax* genomes (Fig. S4). The A. dieselolei B5 genome contained more functional genes than the other four genomes. A. jadensis C13 and C17 genomes did not harbor the genes for periplasmic nitrogen reduction, such as *narGHI*, *nirK*, and *norBZ*, that were present in the other *Alcanivorax* genomes, particularly that of A. dieselolei strain B5 (Fig. S4). Probably nitrate was imported by the NrtABC cross-membrane complex and then was assimilated into amino acids directly by NirBD. Moreover, A. jadensis C13 differed from the other genomes by having *nuoABFKMN* genes, which encode subunits of respiratory chain complex I with proton-pumping function. However, several subunit-coding genes were missing from the genome bin, which leaves a question regarding the proper function of complex I in A. jadensis C13.

### Copies of functional genes in water samples.

**FIG 4:**
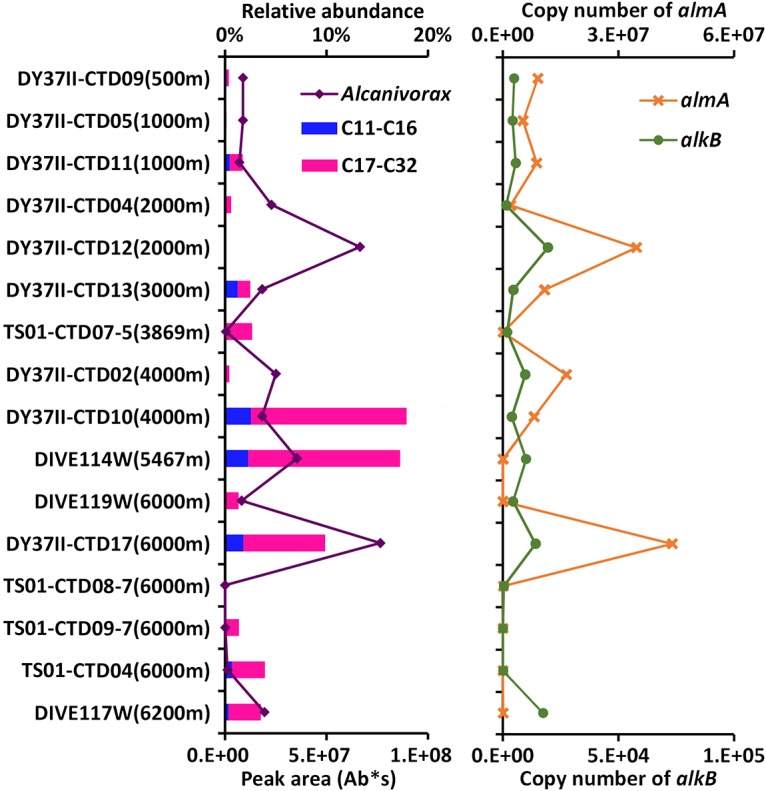
Vertical profiling of *Alcanivorax*, copy numbers of functional genes, and amounts of alkanes in water samples. The abundance of *Alcanivorax* is an estimate based on sequencing of 16S rRNA gene amplicons. The copy numbers of functional *almA* and *alkB* genes of *Alcanivorax* along the water column were inferred from qPCR results. GC-MS was used to detect alkanes in the water samples. Alkanes were categorized into medium-chain (C_11_ to C_16_) and long-chain (C_17_ to C_32_) groups (see Table S2 in the supplemental material for details).

The *almA* and *alkB* genes are the functional genes for alkane degradation by *Alcanivorax*. To validate the association between the prevalence of *Alcanivorax* and the abundance of alkanes, copy numbers of the functional *almA* and *alkB* genes in the samples were examined by quantitative PCR (qPCR). Differences in the community structure of hydrocarbon-degrading microbial populations were assessed via absolute quantification of the genes. A total of 16 samples from different depths and sites were examined. Results showed that the gene copy numbers positively varied with the percentage of *Alcanivorax* (Fig. 4). The samples for DY37II CTD17 at 6,000 m and DY37II CTD12 at 2,000 m harbored the most abundant *almA* and *alkB* genes, which is in accord with the highest percentages of *Alcanivorax* in the microbial communities. There were many more *almA* gene copies than *alkB* gene copies in most of the samples, while copy numbers of *alkB* were higher than those of *almA* in the two bottom waters (DIVE114W and DIVE117W) (Fig. 4).

### Composition of alkanes in water samples.

To explain the spatial distribution pattern of A. jadensis in the Mariana Trench, alkane contents in the water samples were analyzed with gas chromatography-mass spectrometry (GC-MS). From the results, pentadecanoic acid (C_17_) was the most abundant long-chain alkane, followed by hexanedioic acid (C_16_), in all samples. These fatty acids may be used to synthesize the storage lipids in *Alcanivorax* (23). In the DY37II CTD10 sample, there were 10 known alkane species, accounting for the greatest amounts of alkane content (Table S2). The waters from DY37II CTD13 and DY37II CTD17 contained the greatest amounts of long-chain alkanes such as C_19_, C_21_, and C_26_. The results were roughly consistent with the relative abundance of *Alcanivorax* in microbial communities (Fig. 4). DY37II CTD02 and DY37II CTD10 were sampled at the south and north slopes of the trench, respectively. Although both were at a depth of 4,000 m, they differed in the abundance of alkanes. For the two samples from the TS01 cruise, linolelaidic acid was detected in TS01 CTD09-7 (depth of 6,000 m) but not in TS01 CTD08-7 (Fig. 4). The alkane contents in the near-bottom water samples also correlated with the relative abundance of *Alcanivorax* in the community structure. DIVE114W was a bottom water sample collected by a submersible dive from the southern slope of the trench. GC-MS analysis revealed the greatest amount of long-chain (C_32_) alkanes in DIVE114W. TS01 CTD09-7 was close to DY37II CTD17 and at the same sampling depth. The GC-MS results displayed disparities in the contents and abundance of alkanes in the two samples, which was also an indicator of spatial and periodic spreading of alkanes in the Mariana Trench.

### Transcriptional activity.

**FIG 5:**
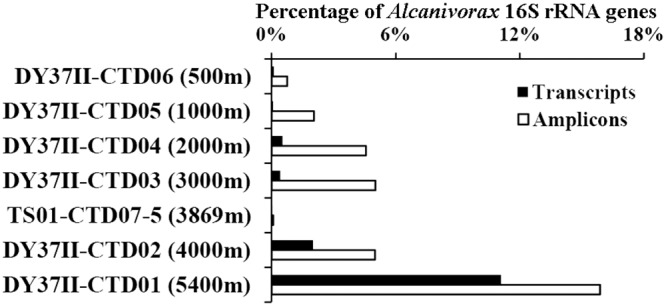
Relative abundance of *Alcanivorax* 16S rRNA in transcripts and amplicons. The 16S rRNA gene transcripts in seven metatranscriptomes were extracted and classified. The percentages assigned to A. jadensis among all of the 16S rRNA gene transcripts are shown. The relative abundance of A. jadensis in the microbial communities of these samples was estimated based on the percentage in sequenced 16S rRNA gene amplicons.

A total of 24 Gbp of metatranscriptomic data for seven aqueous samples was obtained and subjected to quality control. The 16S rRNA genes were then predicted in the data. Those assigned to A. jadensis were the most abundant (11.1%) in the DY37II CTD01 sample, which coincides with the enrichment of A. jadensis in the sample (Fig. 5). The transcriptional level of A. jadensis in the other layers was relatively lower, in agreement with the relative abundance in the communities. The *almA* and *alkB* genes of A. jadensis C13 and C17 were identified in the TS01 CTD07 metatranscriptome (Tables S3 and S4). Totals of 213 and 147 genes were transcribed for A. jadensis C13 and C17, respectively, in the TS01 CTD07 metatranscriptome. The number of the genes identified in the metatranscriptome accounted for ∼6.7% of the gene contents of A. jadensis C13 and C17.

### Global deep sea distribution.

**FIG 6:**
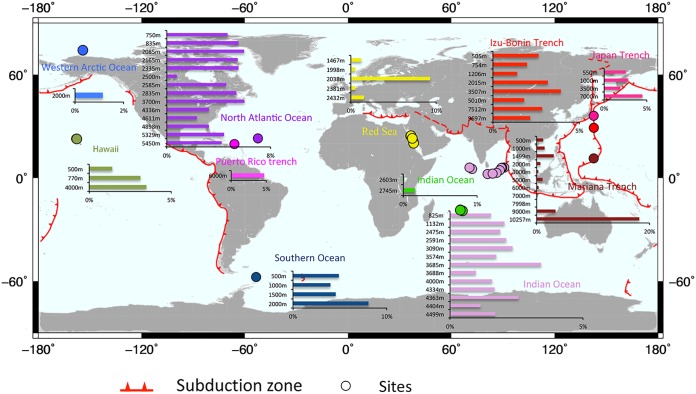
Global distribution of *Alcanivorax* in deep sea layers. The results were based on BLASTN searches of 16S rRNA fragments in 16S rRNA amplicons and metagenomes from public databases. The solid red lines show the locations of subduction zones. The map was created with Generic Mapping Tools (GMT) (66).

Using public data on 16S rRNA gene amplicons and metagenomes, we investigated the distribution of *Alcanivorax* in deep waters of different oceans, particularly along subduction zones (Fig. 6). Overall, *Alcanivorax* was ubiquitous and accounted for less than 5% of the microbial communities in the collected data. At a depth of 10,200 m in the Mariana Trench, *Alcanivorax* accounted for ∼25% of the total. When subduction zones were compared with the other sites, there was not an obviously greater percentage of *Alcanivorax* there. At different depths, several cases of layer-specific *Alcanivorax* richness were observed.

## DISCUSSION

Previous studies on *Alcanivorax* focused on its role in ecological restoration, with very few studies on its spread in deep oceans. Our results showed that it may also be abundant in the depths but not dominant, which is perhaps attributable to the extreme environment of deep seas, with low temperature and high pressure (24). The richness of *Alcanivorax* in certain samples was perhaps due to the large amounts of alkanes released from sediments. In the Mariana Trench, pockmarks were observed by the Jiaolong submersible during the DY37II cruise (Fig. S6). The development of pockmarks is often related to the proliferation of deep hydrocarbon gases (25). A growing body of evidence shows that pockmarks are mainly formed by fluid escaping from under the seabed sediment with the diffusion of oil and gas (26). For the terrain of the Mariana Trench (27), the collision of plates resulted in mass flow from serpentinite-hosted systems, generating hydrocarbons under hydrothermal conditions. In this process, carbon and hydrogen elements accumulate to abiogenically form methane or short-chain and low-molecular-weight alkanes through the interaction of water and rock (28, 29). Undergoing long-term chemical reactions, the short-chain alkanes form medium- and long-chain alkanes. The hydrothermal gases probably fueled the origin of life on Earth (30). In subduction zones such as the Mariana Trench, methane and alkanes might be discharged to the deep sea waters through cracks in rocks and oceanic energy vents (31). Aside from the alkane-producing serpentinization process, alkanes are also generated biologically by organisms, through thermogenesis, and from rotting plants (32, 33). The microbial community structures of the samples collected by the two cruises in this study differed remarkably. *Alcanivorax* species not only were highly abundant in some sites but also were transcriptionally active at different levels in the DY37 samples. The low relative abundance of *Alcanivorax* was consistent with the small amounts of alkanes in the TS01 samples. The unstable supply of alkanes was a possible explanation for the spatial and periodic prevalence of *Alcanivorax*. Along the subduction zones of the world’s oceans, it was not evident that alkane-degrading *Alcanivorax* bloomed in the deep waters, as shown by our results. More sampling sites and time points may contribute to the tight link between approximation to subduction zones and the richness of *Alcanivorax*. In addition, A. jadensis strains C13 and C17 may be considered to be pressure adapted, which is different from previous reports (34, 35). More follow-up studies, such as separations of pure cultures, are required to verify the pressure tolerance.

Degradation of alkanes by *Alcanivorax* is highly dependent on oxygen, which prohibits the spread of *Alcanivorax* in zones and sediments with minimal oxygen (36). In this study, we compared the microbial community structures in bottom water samples and sediments. The dramatic difference in *Alcanivorax* abundance was probably attributable to the low oxygen flux in the sediments. In this regard, alkanes likely could be consumed only in waters. The slow degradation of long-chain alkanes may spread long distances with the effects of deep seawater currents. It was reported that, with the effects of physicochemical factors, spilled oil formed an oil plume containing gaseous and monoaromatic compounds, and a hydrocarbon-dependent microbial community in the plume was proposed (34). However, our study did not detect *Colwellia*, *Cycloclasticus*, and *Pseudoalteromonas*, which may multiply to form dominant bacterial groups in the plumes (34). Catching the plume is technically difficult, because it is rather dynamic under the influence of the water flow. This may partially explain the incongruent GC-MS and *Alcanivorax* relative abundance results, as well as the lack of consistency between the distribution of subduction zones and the relative abundance of *Alcanivorax*.

Although the related regulatory genes were identified in *Alcanivorax* genomes, the expression-regulating mechanism was unknown (37, 38). The presence of the three known alkane-degrading pathways indicates that A. jadensis in the present study might utilize alkanes of different sizes. Strains with the *alkB* gene were abundant in the bottom waters, probably due to the rapid consumption of short- and medium-chain alkanes. In the water column, the *almA* genes outcompeted the *alkB* genes in copy number, indicating that the *Alcanivorax* inhabitants there were prone to degradation of medium- and long-chain alkanes. This allows the proposition that the alkanes were released from the sediments and small alkanes were degraded first by the *Alcanivorax* strains bearing more *alkB* genes in the bottom waters.

Most alkane-degrading bacteria, including *Colwellia*, *Cycloclasticus*, and *Pseudoalteromonas*, were reported in shallow and coastal waters (39). In deep waters of open oceans, *Alcanivorax* is the predominant species for the degradation of alkanes of various sizes (40). The degradation of long-chain alkanes may also provide a carbon source for heterotrophic bacteria in the deep sea. In the microbial communities revealed for the DY37 cruise, the richness of *Alcanivorax* was associated with that of *Alteromonas*, a heterotrophic bacterium (41) (see Fig. S5 in the supplemental material). In contrast, there was distinct richness of *Novosphingobium* and *Sphingobium* in the samples collected by the TS01 cruise. The prevalence of polycyclic aromatic hydrocarbon (PAH) degraders (42) indicates a nutrient-poor deep sea environment at the TS01 sampling sites. The assimilatory nitrate reduction process carried out by the deep sea A. jadensis strains in this study likely could rapidly produce organic nitrogen sources such as amino acids and vitamins, which are important for the development and maintenance of deep sea ecosystems. There is some dissimilatory nitrate reduction in other *Alcanivorax* species, but the A. jadensis strains in this study seem to be independent of the periplasmic nitrate as an election acceptor. This was perhaps accounted for by sufficient oxygen flux for the deep inhabitants in the water column. A previous study reported a dissolved oxygen concentration of 172 μM at a depth of 6,000 m in the Mariana Trench (24), which allows rapid growth of *Alcanivorax*.

How the three alkane-degrading pathways are regulated in *Alcanivorax* is still an open question at present. More experiments, *in situ* cultivation, and transcriptomic work may provide clues to the puzzle. In sum, the microbial communities are more dynamic than previously expected, due to rapid changes in deep sea environments. Sampling efforts at more sites, time points, and depths with advanced techniques will cast light on the metabolic activities and adaptation strategies of deep sea microbial lineages with important ecological contributions.

## MATERIALS AND METHODS

### Sample collection and nucleotide extraction.

Two research cruises were carried out in the Mariana Trench in the summer of 2016, i.e., DY37II (from 4 June to 13 July 2016) and TS01 (from 22 June to 12 August 2016). About 70 liters of water was obtained for each sample, at different depths and sites, with Niskin bottles on the CTD sensors (Fig. 1A; also see Table S1 in the supplemental material). Push cores and 10-liter samples of bottom waters were also obtained, by four Jiaolong submersible dives (Fig. 1A). Water samples were filtered through 0.22-μm polycarbonate membranes (Millipore, Bedford, MA, USA). After filtration, the membranes were immediately frozen at −80°C for metagenomic work, and 500-ml samples of filtered water were stored at 4°C for chemical component analysis. A thin layer of the push cores was removed, and the remaining sediment was sliced into 2-cm layers.

DNA was extracted from the polycarbonate membranes and sediment layers from 0 to 2 cm below the seafloor (cmbsf) using the Mo Bio PowerSoil DNA isolation kit (Mo Bio, Carlsbad, CA, USA). RNA was extracted using the Mo Bio PowerSoil total RNA isolation kit, and DNA was degraded with the Turbo DNA-free kit (Ambion, Carlsbad, CA, USA). Reverse transcription was completed with the Ovation RNA-sequencing system v2 kit (NuGEN, San Carlos, CA, USA). Nucleotide concentrations were estimated with a Qubit 2.0 fluorometer (Invitrogen, Carlsbad, CA, USA). The quality of nucleotides was checked with gel electrophoresis.

### Sequencing of 16S rRNA gene amplicons and data analyses.

A pair of universal primers (U341F, 5′-CCTAYGGGRBGCASCAG-3′; U802R, 5′-TACNVGGGTATCTAATCC-3′) was used to amplify the V3 to V4 region of the 16S rRNA gene, in triplicate (43). Six-nucleotide barcodes were appended to the primers for subsequent separation of different samples. The PCR was prepared according to the protocol for PrimeSTAR HS DNA polymerase (TaKaRa, Dalian, China), with 2 μl of forward and reverse primers (10 μM) and 1 ng of template DNA. The PCR was performed on a thermal cycler (Bio-Rad, Hercules, CA, USA), with the following program: (i) 10 s at 98°C to unwind the DNA, (ii) 30 cycles of 10 s at 98°C, 15 s at 50°C, and 30 s at 72°C, and (iii) 5 min at 72°C for extension. The quality of the PCR products was checked with gel electrophoresis, and the products were purified using the Cycle Pure kit (Omega, Norcross, GA, USA). All of the PCR products were mixed together, in the same amounts, for preparation of the Illumina sequencing library. The amplicons were sequenced on the Illumina MiSeq platform.

The adaptors, low-quality reads, and ambiguous nucleotides were trimmed from the raw data with the NGS QC Toolkit (44). The quality-filtered reads were assigned to OTUs at a 97% similarity level with UCLUST (45). The longest read of each OTU was selected as the representative for subsequent taxonomic classification. Taxonomic assignment was conducted using the Ribosomal Database Project (RDP) classifier v2.2 (46), by referring to the Silva128 database (47), with a confidence level of 80%. Calculation of diversity indices and OTU clustering were performed with QIIME v1.9.1 (48).

### Metagenomic analyses.

A total of 200 ng of genomic DNA was fragmented to ∼500 bp by ultrasonication. Genomic libraries were arranged using the TruSeq Nano DNA Library kit (Illumina). The final library containing all of the pooled samples was sequenced on the MiSeq platform (Illumina). The raw data were evaluated using FastQC (49); low-quality reads that included N or were shorter than 50 bp were excluded. The quality of the paired-end reads (3 Gbp) was assessed using the NGS QC Toolkit (44), which resulted in filtration of low-quality reads with a minimum value of Q20. Clean data were assembled into scaffolds by SPAdes v3.6.2 (50). Genome binning was accomplished as reported previously (51). The read coverage and G+C contents of the contigs were used for binning of the genome bins, followed by a correspondence analysis of the tetranucleotide frequencies of their respective contigs (51). The 16S rRNA genes in the contigs were identified using rRNA_HMM (52). The completeness and contamination rate were assessed by CheckM v1.0.5 (53).

Coding sequences (CDSs) and proteins in the binned genomes were predicted by Prodigal v2.6.2 (54). Annotation of CDSs was carried out via BLASTP against databases including the NCBI nonredundant protein database, the KEGG database (55), and the Clusters of Orthologous Groups (COG) database (56), with a cutoff E-value of 1e−05.

### Metatranscriptome sequencing and data analysis.

cDNA libraries were prepared with the Ovation Ultralow v2 1–16 kit (NuGEN, San Carlos, CA, USA) and sequenced on the MiSeq platform (Illumina). The raw data were evaluated using FastQC (49). The 16S rRNA genes in the metatranscriptomic data were predicted by SortMeRNA v2.1 (57) and classified by referring to the Silva128 database (47), with a confidence level of 90%. The quality-filtered reads were assembly by Trinity v2.3.2 (58). The genes that resembled the open reading frames (ORFs) predicted for the A. jadensis genome bins were identified by BLASTN, with a sequence identity threshold value of 90% (59).

### Phylogenetic and correlation analyses.

The 16S RNA genes were pooled with the representative OTUs assigned to *Alcanivorax* and those of the relatives identified in the NCBI nucleotide database. All of the sequences were aligned with DNAMAN v6.0 (Lynnon Biosoft, San Ramon, CA, USA) and adjusted manually with MEGA v5.0 (60). Using a maximum-likelihood algorithm, a phylogenetic tree was constructed using raxmlGUI v1.5, with 1,000 replicates (61). The regions with homology between the binned genomes and the references were detected by a pairwise BLASTn search. The homologous regions were displayed by Circos v0.69-2 (62).

### GC-MS analysis of hydrocarbons.

The water samples filtered with 0.22-μm polycarbonate membranes (Millipore) were shaken gently and extracted twice with 100 ml of methylene chloride and subsequently with 100 ml of hexane (63). The extracts was mixed and rotary-evaporated to 1 ml. A column filled with activated silica gel (mesh size of 200 to 325) was used for chromatography of the hexane extract. Twenty milliliters of hexane/methylene chloride (70:30 [vol/vol]) was used to elute the hydrocarbons (64). The collected distillate was blown dry with a nitrogen evaporator (Allsheng, Hangzhou, China). Before being injected into an Agilent 7890A/5975C GC-MS system (Agilent, Santa Clara, CA, USA), the samples were dissolved with 100 μl hexane. The resulting patterns were searched in the NIST14.L library. The area of the peaks was calculated as an estimate of amount of certain alkane content. The chemical components present in blank control that possibly resulted from containers, environment and solutions were then excluded from the GC-MS results of the water samples.

### Quantification of functional genes.

Two pairs of primers were designed for the *almA* and *alkB* genes (almA-F, 5′-CGTGACAACGAAGACTGCATTAC-3′; almA-R, 5′-CGTGACAACGAAGACTGCATTAC-3′; alkB-F, 5′-TTGCGTTTGAAAAAGTGGGG-3′; alkB-R, 5′-CGGTAGCTCTTGTCCTGGGA-3′), based on the gene predictions for the genome. PCR amplification of the genes was performed with the 2× *Taq* PCR Master Mix kit (TianGen, Dalian, China). The fragments were cloned into the pMD18-T vector (TaKaRa) and then transformed into competent JM109 cells (TaKaRa). Colony PCR was performed by direct PCR with the M13F and M13R primers. After bacterial culture, the plasmid was extracted using a plasmid midi kit (Omega, Norcross, GA, USA). The DNA was serially diluted 10-fold, as were the standard PCR products. The gene copy numbers were estimated by qPCR in triplicate, using the StepOnePlus real-time PCR system (Applied Biosystems, Foster City, CA, USA) with SYBR green I (TaKaRa). The qPCR was performed in a 20-μl reaction mixture with 2 μl of template DNA (1 ng), 0.8 μl of each primer (10 nM), 0.4 μl of ROX reference dye, and 6 μl of sterile water. The thermal cycling conditions were 95°C for 30 s, 40 cycles of 95°C for 5 s and 60°C for 30 s, and a melting curve stage of 95°C for 15 s, 60°C for 1 min, and 95°C for 15 s.

### Data availability.

Data supporting the results of this article have been deposited in GenBank under BioProject no. PRJNA421240. The genome accession numbers are QAZC00000000 and QAZB00000000. SRA accession of sequence reads for 16S rRNA amplicons are SRR6466501, SRR6466502, SRR6466503, and SRR6466504.

## Supplementary Material

Supplemental file 1
