# Supplementary material for: Periodic and Spatial Spreading of Alkanes and Alcanivorax Bacteria in Deep Waters of the Mariana Trench
Source: Appl Environ Microbiol. 2019 Jan 23;85(3):e02089-18. doi: 10.1128/AEM.02089-18 (PMC6344633; doi:10.1128/AEM.02089-18)
Supplement: Supplemental file 1 [file fa50710dfa93519b24061dadd9a6d965_AEM.02089-18-s0001.pdf]

Table S1. Samples and percentage of *Alcanivorax* in the communities.

Cruise	Sample ID (Temperature ℃)	Longitude [degrees East]	Latitude [degrees North]	Depth [m]	Date	*Alcanivorax* (%)
DY37II	DY37II-CTD01 (1.52)	141.924	10.870	5400	2016.06.10	16.3
DY37II	DY37II-CTD02	141.893	10.853	4000	2016.06.12	5.1
DY37II	DY37II-CTD03 (1.68)	141.952	10.847	3000	2016.06.13	5.1
DY37II	DY37II-CTD04 (2.31)	141.943	10.849	2000	2016.06.14	4.5
DY37II	DY37II-CTD05 (4.74)	141.899	10.865	1000	2016.06.14	2.0
DY37II	DY37II-CTD06 (7.16)	141.870	10.875	500	2016.06.14	1.1
DY37II	DY37II-CTD07-1	141.834	10.882	1000	2016.06.14	0.7
DY37II	DY37II-CTD07-2	141.834	10.882	2000	2016.06.14	3.4
DY37II	DY37II-CTD07-3	141.834	10.882	3000	2016.06.14	17.8
DY37II	DY37II-CTD07-4	141.834	10.882	4000	2016.06.14	12.0
DY37II	DY37II-CTD07-5	141.834	10.882	4600	2016.06.14	3.6
DY37II	DY37II-CTD08 (1.47)	141.658	10.849	4500	2016.06.15	8.9
DY37II	DY37II-CTD09 (7.18)	141.988	10.940	500	2016.06.17	1.6
DY37II	DY37II-CTD10	141.982	11.763	4000	2016.06.19	3.5
DY37II	DY37II-CTD11 (4.87)	141.983	11.545	1000	2016.06.19	1.3
DY37II	DY37II-CTD12	141.996	11.548	2000	2016.06.19	12.2
DY37II	DY37II-CTD13	141.900	11.526	3000	2016.06.20	3.4
DY37II	DY37II-CTD17	141.985	11.559	6000	2016.06.25	14.8
TS01	TS01-CTD02-1(7.43)	141.500	11.800	500	2016.06.29	0.2
TS01	TS01-CTD02-2(4.79)	141.500	11.800	1000	2016.06.29	0.2
TS01	TS01-CTD02-3(2.33)	141.500	11.800	2000	2016.06.29	0.2
TS01	TS01-CTD02-4(1.68)	141.500	11.800	3000	2016.06.29	2.8
TS01	TS01-CTD03-1(7.5)	141.500	11.600	500	2016.06.30	0.1
TS01	TS01-CTD03-2(4.79)	141.500	11.600	1000	2016.06.30	0.2
TS01	TS01-CTD03-3(2.34)	141.500	11.600	2000	2016.06.30	0.1
TS01	TS01-CTD03-4(1.71)	141.500	11.600	3000	2016.06.30	0.5
TS01	TS01-CTD03-5(1.5)	141.500	11.600	4000	2016.06.30	0.9
TS01	TS01-CTD03-6(1.43)	141.500	11.600	4590	2016.06.30	0.1
TS01	TS01-CTD04(1.6)	142.017	10.950	6011	2016.07.02	0.2
TS01	TS01-CTD06-1(4.66)	141.500	10.150	1000	2016.07.05	0.3
TS01	TS01-CTD06-2(2.29)	141.500	10.150	2000	2016.07.05	0
TS01	TS01-CTD06-3(1.68)	141.500	10.150	3000	2016.07.05	0.2
TS01	TS01-CTD06-4(1.5)	141.500	10.150	3700	2016.07.05	0.1
TS01	TS01-CTD07-1(7.13)	141.500	10.400	500	2016.07.06	0.1
TS01	TS01-CTD07-2(4.69)	141.500	10.400	1000	2016.07.06	0.6
TS01	TS01-CTD07-3(2.17)	141.500	10.400	2000	2016.07.06	0
TS01	TS01-CTD07-4(1.69)	141.500	10.400	3000	2016.07.06	0
TS01	TS01-CTD07-5(1.47)	141.500	10.400	3869	2016.07.06	0.9
TS01	TS01-CTD08-1(7.11)	141.500	10.900	500	2016.07.07	0.1

TS01	TS01-CTD08-2(4.62)	141.500	10.900	1000	2016.07.07	0.1
TS01	TS01-CTD08-3(2.27)	141.500	10.900	2000	2016.08.01	0.1
TS01	TS01-CTD08-4(1.68)	141.500	10.900	3000	2016.08.01	2.3
TS01	TS01-CTD08-5(1.52)	141.500	10.900	4000	2016.08.01	0
TS01	TS01-CTD08-6(1.47)	141.500	10.900	5000	2016.07.07	0
TS01	TS01-CTD08-7(1.6)	141.500	10.900	6010	2016.07.07	0.1
TS01	TS01-CTD09-1(7.22)	141.500	11.400	500	2016.07.11	0.1
TS01	TS01-CTD09-2(4.7)	141.500	11.400	1000	2016.07.11	0.1
TS01	TS01-CTD09-3(2.27)	141.500	11.400	2000	2016.07.11	0
TS01	TS01-CTD09-4(1.66)	141.500	11.400	3000	2016.07.11	0.2
TS01	TS01-CTD09-5(1.51)	141.500	11.400	4000	2016.07.11	0.1
TS01	TS01-CTD09-6(1.48)	141.500	11.400	5000	2016.07.11	0.1
TS01	TS01-CTD09-7(1.6)	141.500	11.400	6000	2016.07.11	0.1
TS01	TS01-CTD10-1(6.92)	141.500	11.150	500	2016.07.12	0
TS01	TS01-CTD10-2(4.52)	141.500	11.150	1000	2016.07.12	0.1
TS01	TS01-CTD10-3(2.3)	141.500	11.150	2000	2016.07.12	0.1
TS01	TS01-CTD10-4(1.69)	141.500	11.150	3000	2016.07.12	0.1
TS01	TS01-CTD10-5(1.52)	141.500	11.150	4000	2016.07.12	0
TS01	TS01-CTD10-6(1.47)	141.500	11.150	5000	2016.07.12	0.2
TS01	TS01-CTD10-7(1.6)	141.500	11.150	6000	2016.07.12	0.1
TS01	TS01-CTD11-1(7.26)	141.500	10.650	500	2016.07.13	0
TS01	TS01-CTD11-2(4.83)	141.500	10.650	1000	2016.07.13	0.1
TS01	TS01-CTD11-3(2.28)	141.500	10.650	2000	2016.07.13	0
TS01	TS01-CTD11-4(1.67)	141.500	10.650	3000	2016.07.13	0
TS01	TS01-CTD11-5(1.45)	141.500	10.650	4653	2016.07.13	0
TS01	TS01-CTD12(1.72)	141.839	11.208	6802	2016.07.16	0
TS01	TS01-CTD13(1.72)	141.699	11.221	6802	2016.07.16	0.1
TS01	TS01-CTD14(1.72)	142.191	11.293	6802	2016.07.20	0.2
DY37II	DIVE114S(1.53)	141.953	10.850	5467	2016.06.11	0
DY37II	DIVE114W(1.53)	141.953	10.850	5467	2016.06.11	7.1
DY37II	DIVE116S(1.67)	141.939	10.950	6500	2016.06.16	0.1
DY37II	DIVE116W(1.67)	141.939	10.950	6500	2016.06.16	5.3
DY37II	DIVE117W(1.62)	142.248	11.665	6200	2016.06.18	3.9
DY37II	DIVE119S(1.6)	142.202	10.885	6000	20160.6.24	0
DY37II	DIVE119W(1.6)	142.202	10.885	6000	20160.6.24	1.6

Table S2. Detection of hydrocarbons and oxidised derivatives. We used the peak area of the spectrum as an indicator of abundance of alkanes. The samples are referred to Table S1.

	DY37II-CTD09(500m)	DY37II-CTD05(1000m)	DY37II-CTD11(1000)	DY37II-CTD04(2000m)	DY37II-CTD12(2000m)	DY37II-CTD13(3000m)	TS01-CTD07-5(3869m)	DY37II-CTD02(4000m)	DY37II-CTD10(4000m)	DIVE114W(5467m)	DIVE119W(6000m)	DY37II-CTD17(6000m)	TS01-CTD08-7(6000m)	TS01-CTD09-7(6000m)	TS01-CTD04(6011m)	DIVE117W(6200m)
Nonane, 4,5-dimethyl- (C11)										2880818					925977	
2-Bromo dodecane (C12)						5181658				590327					287055	
Dodecane, 1-fluoro-(C12)										428339						
1-Iodo-2-methylundecane(C12)									1398007							
Dodecane, 1-iodo-(C12)												8715609				
Nonane, 5-(1-methylpropyl)-(C13)										372031						
Tridecane(C13)			1037292													
Undecane, 2,3-dimethyl-(C13)						1095100										
Decane, 2,3,6-trimethyl-(C13)												2149858				
Tetradecane(C14)									1267762	502756						
Methyl tetradecanoate(C15)									1442288							
Dodecane, 2,6,10-trimethyl-(C15)											202986					
7-Hexadecyne(C16)									4239099							
Hexadecane, 1-iodo-(C16)																2144999
Hexadecane(C16)	981757		1774077			1322822			7040274	4295526					2103054	
Nonane, 2,2,4,4,6,8,8-heptamethyl-(C16)										1076767	385492					
1-Decanol, 2-hexyl-(C16)										3611554					784123	
Hexadecanoic acid, methyl ester(C17)				3656132			4540106									6319962
Pentadecanoic acid, 14-methyl-, methyl ester(C17)	1372987	522362						2553238	41832512			2441348				

Tetradecane, 2,6,10-trimethyl-(C17)									1950666	325013				831964	1105659
Heptadecane(C17)						1003866						2573524				
Linoelaidic acid(C18)														8342705		
cis-9-Tetradecenoic acid, isobutyl ester											125712				217382	
Z-10-Methyl-11-tetradecen-1-ol propionate(C18)											194906				244730	
cis-1-Chloro-9-octadecene(C18)										1399953						
5-Octadecene, (E)-(C18)									4657881							
Octadecane, 1-chloro-(C18)										533333	118128				721980	
Octadecane, 1-iodo-(C18)							929986			1182783	936093				2772394	2095795
Octadecane(C18)										4351137						
Heptadecane, 8-methyl-(C18)									8355534							
12-Methyl-E,E-2,13-octadecadien-1-ol(C19)									1105578							
trans-13-Octadecenoic acid, methyl ester(C19)									3959561							
Heptadecanoic acid, 16-methyl-, methyl ester(C19)							1074419									1623011
Nonadecane(C19)						1727272						1837515				
2-Methyl-cis-7,8-epoxynonadecane(C20)										1316502						
Hexadecane, 2,6,10,14-tetramethyl-(C20)										13376244	1587215				3227795	1351973
Hexadecane, 2,6,11,15-tetramethyl-(C20)										3251821					2979111	
Eicosane(C20)									8054669	4300819					586043	
2,2-Dimethyloctadecane(C20)															416402	
10-Methylnonadecane(C20)		108983	2600222													

Heptadecane, 2,6,10,15-tetramethyl-(C21)									2458020		155233	909959				
Heneicosane(C21)						2738113			5370372	7095884		7644253				
1-Heneicosanol(C21)									10482863							
Batilol(C21)											119973					
Z-5-Methyl-6-heneicosen-11-one(C22)										338851						
Docosane(C22)							2236432			5881896						
2,2-Dimethyleicosane(C22)															344499	
Carbonic acid, eicosyl vinyl ester(C23)										3048239		1600859				
1-Tricosanol(C23)										2110759						
Eicosyl heptafluorobutyrate(C24)										2328608						
Tetracosane(C24)										7172393					919942	
Undec-10-ynoic acid, tetradecyl ester																
Pentacosane(C25)							2733322			4259478	1177421				1604960	1908530
2-Methyltetracosane(C25)			2496961									1039566				
Octadecane, 3-ethyl-5-(2-ethylbutyl)-(C26)										810648	581365				239315	1141565
Hexacosane(C26)		106854				1873487						11134558				
Carbonic acid, decyl hexadecyl ester(C27)										3248177						
2-Methylhexacosane(C27)										992078	237453					
Heptacosane(C27)		98960					2966146			4251929	386603				661354	
Octacosane(C28)		21117	2508289									10463088				
2-methyloctacosane(C29)												1979557				
Triacontane(C30)					610755											
Hentriacontane(C31)									5705307	5014407						
Dotriacontane, 1-iodo-(C32)							1595185			11568871	1481571	6707141			3779646	3527003

Table S3. Transcripts of *Alcanivorax jadensis* C17 in the metatranscriptomes.

Transcriptome	Contig ID in Transcriptome	*A. jadensis* C17 gene ID	Annotation
DY37-CTD01	TRINITY_DN203_c0_g1_i2_2	26_98	50S ribosomal protein L2
	TRINITY_DN1338_c0_g1_i1_1	12_46	glutamyl-tRNA amidotransferase
DY37-CTD02	-	-	-
DY37-CTD03	TRINITY_DN509_c0_g1_i1_1	120_27	bifunctional prephenate dehydrogenase/3-phosphoshikimate 1-carboxyvinyltransferase
	TRINITY_DN0_c0_g1_i1_1	26_98	50S ribosomal protein L2
DY37-CTD04		-	-
DY37-CTD05	TRINITY_DN1656_c0_g1_i1_1	387_1	cytochrome B
DY37-CTD06	TRINITY_DN1087_c0_g1_i1_1	130_14	cytochrome c oxidase assembly protein
	TRINITY_DN1201_c0_g1_i1_1	120_27	bifunctional prephenate dehydrogenase/3-phosphoshikimate 1-carboxyvinyltransferase
TS01-CTD07-5	TRINITY_DN235232_c0_g1_i2_1	107_10	radical SAM/Cys-rich domain protein
	TRINITY_DN143182_c0_g1_i1_1	107_5	DNA mismatch repair protein MutS
	TRINITY_DN169630_c0_g1_i1_1	113_12	citrate synthase/methylcitrate synthase
	TRINITY_DN347134_c0_g1_i1_1	113_9	putative methylaconitate Delta-isomerase PrpF
	TRINITY_DN153548_c0_g1_i1_1	114_2	50S ribosomal protein L25/general stress protein Ctc
	TRINITY_DN168952_c0_g1_i1_1	114_3	ribose-phosphate pyrophosphokinase
	TRINITY_DN184011_c0_g1_i1_2	114_38	RNA polymerase factor sigma-54
	TRINITY_DN448109_c0_g1_i1_1	117_13	cysteine desulfurase
	TRINITY_DN571068_c0_g1_i1_1	12_44	DNA primase
	TRINITY_DN193839_c0_g3_i1_1	12_48	4'-phosphopantetheinyl transferase
	TRINITY_DN400121_c0_g1_i1_1	12_67	acyl-CoA dehydrogenase
	TRINITY_DN285341_c0_g1_i1_1	120_27	bifunctional prephenate dehydrogenase/3-phosphoshikimate 1-carboxyvinyltransferase
	TRINITY_DN301966_c0_g1_i1_1	120_33	3-demethylubiquinone-9 3-methyltransferase
	TRINITY_DN212486_c0_g3_i1_1	122_4	glycine dehydrogenase (aminomethyl-transferring), partial

TRINITY_DN358538_c0_g1_i1_1	125_27	NADH:ubiquinone reductase (Na(+)-transporting) subunit D
TRINITY_DN8030_c0_g1_i1_1	130_14	cytochrome c oxidase assembly protein
TRINITY_DN404282_c0_g1_i1_1	130_19	transcriptional regulator
TRINITY_DN224335_c0_g3_i1_1	133_21	XRE family transcriptional regulator
TRINITY_DN71274_c0_g1_i1_1	134_8	DNA topoisomerase IV subunit B
TRINITY_DN201910_c0_g1_i1_1	134_9	DNA topoisomerase IV subunit A
TRINITY_DN167802_c0_g2_i1_1	135_22	peptide chain release factor 3
TRINITY_DN183936_c0_g1_i1_1	15_122	methionyl-tRNA formyltransferase
TRINITY_DN173611_c0_g1_i1_1	15_39	cell division ATP-binding protein FtsE
TRINITY_DN515931_c0_g1_i1_1	15_81	ribonuclease PH
TRINITY_DN310345_c0_g1_i1_1	15_85	dihydroxy-acid dehydratase
TRINITY_DN232554_c0_g2_i1_1	15_88	(p)ppGpp synthetase
TRINITY_DN191147_c1_g1_i1_1	151_15	aspartate alpha-decarboxylase
TRINITY_DN93534_c0_g1_i1_1	158_11	electron transfer flavoprotein subunit beta
TRINITY_DN164157_c0_g2_i1_1	158_12	electron transfer flavoprotein subunit beta
TRINITY_DN330674_c0_g1_i1_1	169_11	UDP-N-acetylmuramate--L-alanine ligase
TRINITY_DN186736_c0_g1_i1_1	170_18	methylcrotonoyl-CoA carboxylase
TRINITY_DN172336_c0_g1_i1_1	170_22	MerR family transcriptional regulator
TRINITY_DN436498_c0_g1_i1_1	175_10	hypothetical protein
TRINITY_DN182079_c0_g1_i1_1	175_22	peptidase M23
TRINITY_DN329540_c0_g1_i1_1	178_11	succinate--CoA ligase subunit beta
TRINITY_DN518378_c0_g1_i1_1	178_20	gluconolactonase, partial
TRINITY_DN224441_c0_g1_i1_1	18_107	carbamoyl phosphate synthase small subunit
TRINITY_DN294193_c0_g2_i1_1	18_108	Carbamoyl-phosphate synthase large chain
TRINITY_DN364200_c0_g1_i1_1	18_59	elongation factor P
TRINITY_DN274771_c0_g1_i1_1	18_94	molecular chaperone DnaK
TRINITY_DN273179_c0_g1_i1_1	18_95	molecular chaperone DnaJ
TRINITY_DN530913_c0_g1_i1_1	199_8	acyl-CoA dehydrogenase
TRINITY_DN519803_c0_g1_i1_1	208_2	type IV-A pilus assembly ATPase PilB
TRINITY_DN543762_c1_g1_i1_1	231_1	elongation factor Tu

TRINITY_DN530618_c0_g1_i1_2	231_2	elongation factor G
TRINITY_DN173758_c0_g1_i1_1	231_5	DNA-directed RNA polymerase subunit beta'
TRINITY_DN111891_c0_g1_i1_1	231_6	DNA-directed RNA polymerase subunit beta
TRINITY_DN233851_c0_g2_i1_1	25_17	tRNA uridine-5-carboxymethylaminomethyl(34) synthesis enzyme MnmG
TRINITY_DN464907_c0_g1_i1_1	25_2	glutamine--fructose-6-phosphate aminotransferase
TRINITY_DN195640_c0_g1_i1_1	25_3	UDP-N-acetylglucosamine diphosphorylase/glucosamine-1-phosphate N-acetyltransferase
TRINITY_DN139945_c0_g1_i1_1	25_5	F0F1 ATP synthase subunit beta
TRINITY_DN193314_c0_g1_i1_1	25_50	hypothetical protein
TRINITY_DN195215_c0_g1_i1_1	25_76	isomerase/hydrolase
TRINITY_DN367172_c0_g1_i1_1	26_103	elongation factor Tu
TRINITY_DN149908_c1_g1_i1_1	26_13	NAD+ synthase
TRINITY_DN323425_c0_g1_i1_2	26_25	4-hydroxy-3-methylbut-2-enyl diphosphate reductase
TRINITY_DN224675_c0_g2_i1_1	26_28	isoleucine--tRNA ligase
TRINITY_DN168467_c0_g2_i1_1	26_42	short chain dehydrogenase
TRINITY_DN469651_c0_g1_i1_1	26_81	preprotein translocase subunit SecY
TRINITY_DN333729_c0_g1_i1_1	26_89	50S ribosomal protein L5
TRINITY_DN232442_c0_g4_i1_1	26_9	ATP-dependent chaperone ClpB
TRINITY_DN151558_c0_g2_i1_1	26_94	50S ribosomal protein L16
TRINITY_DN542343_c0_g1_i1_1	27_45	ATP-dependent RNA helicase DbpA
TRINITY_DN238543_c1_g1_i1_1	27_80	serine peptidase
TRINITY_DN272025_c0_g1_i1_1	27_88	hybrid sensor histidine kinase/response regulator
TRINITY_DN524468_c0_g1_i1_2	287_2	glutamate-5-semialdehyde dehydrogenase
TRINITY_DN111826_c0_g1_i1_1	29_43	DNA-binding protein
TRINITY_DN509335_c0_g1_i1_1	29_46	translation initiation factor IF-1
TRINITY_DN186359_c0_g1_i1_1	29_54	serine--tRNA ligase
TRINITY_DN230719_c0_g1_i1_1	29_56	glycosyl transferase
TRINITY_DN106482_c0_g2_i1_2	29_64	carbon starvation protein A
TRINITY_DN354141_c0_g1_i1_1	29_74	type II secretion system protein GspF
TRINITY_DN219286_c0_g1_i1_1	29_89	hypothetical protein

TRINITY_DN520113_c0_g1_i1_1	31_1	acyl-CoA dehydrogenase, partial
TRINITY_DN403199_c0_g1_i1_1	31_12	acyl-CoA dehydrogenase
TRINITY_DN530645_c0_g1_i1_1	31_25	hypothetical protein
TRINITY_DN126417_c0_g1_i1_1	31_34	aldehyde dehydrogenase
TRINITY_DN556115_c0_g1_i1_1	312_12	monovalent cation/H+ antiporter subunit D
TRINITY_DN70368_c0_g1_i1_1	33_30	tryptophan synthase subunit beta
TRINITY_DN324782_c0_g1_i1_1	33_55	ATP-dependent Clp protease ATP-binding subunit ClpX
TRINITY_DN175259_c0_g1_i1_1	33_56	endopeptidase La
TRINITY_DN239794_c0_g1_i1_1	33_60	ABC transporter ATP-binding protein
TRINITY_DN453910_c0_g1_i1_1	33_62	microcin ABC transporter permease
TRINITY_DN239306_c0_g1_i1_1	33_82	ABC1 family protein, ubiquinone biosynthesis protein
TRINITY_DN235328_c0_g1_i1_1	34_31	AAA family ATPase
TRINITY_DN225468_c0_g1_i1_1	34_83	AraC family transcriptional regulator
TRINITY_DN219083_c0_g1_i1_1	37_4	acetyl-coenzyme A synthetase
TRINITY_DN233885_c0_g1_i1_2	37_61	30S ribosomal protein S6
TRINITY_DN397681_c0_g1_i1_1	37_71	adenylosuccinate synthase
TRINITY_DN398813_c0_g1_i1_1	387_1	cytochrome B
TRINITY_DN325258_c0_g1_i1_1	469_1	hybrid sensor histidine kinase/response regulator
TRINITY_DN438986_c0_g1_i1_1	47_3	class II aldolase
TRINITY_DN305499_c0_g1_i1_1	47_6	hybrid sensor histidine kinase/response regulator
TRINITY_DN231767_c0_g5_i1_1	47_60	hypothetical protein
TRINITY_DN339105_c0_g1_i1_1	47_9	NAD-glutamate dehydrogenase
TRINITY_DN240389_c2_g8_i1_1	49_2	acetolactate synthase
TRINITY_DN194388_c0_g1_i1_1	49_22	aconitate hydratase
TRINITY_DN197518_c0_g1_i1_1	53_18	argininosuccinate lyase
TRINITY_DN486274_c0_g1_i1_1	53_4	dihydroxy-acid dehydratase
TRINITY_DN199200_c0_g4_i1_1	53_49	excinuclease ABC subunit B
TRINITY_DN24748_c0_g1_i1_1	56_17	proline--tRNA ligase
TRINITY_DN226172_c0_g1_i1_1	56_23	ATP-dependent DNA helicase RecQ
TRINITY_DN238251_c1_g4_i1_1	56_26	RNA helicase

TRINITY_DN450296_c0_g1_i1_1	56_56	ribonucleotide-diphosphate reductase subunit beta
TRINITY_DN161529_c0_g1_i1_1	59_36	alanine--tRNA ligase
TRINITY_DN464120_c0_g2_i1_1	59_53	glyceraldehyde 3-phosphate dehydrogenase
TRINITY_DN257439_c0_g1_i1_1	60_26	arginine--tRNA ligase
TRINITY_DN106945_c0_g1_i1_1	60_27	primosomal protein N'
TRINITY_DN198893_c0_g3_i1_1	60_29	malate dehydrogenase
TRINITY_DN389716_c0_g1_i1_1	60_30	peptidase
TRINITY_DN185810_c1_g1_i1_1	60_40	glutamate synthase large subunit
TRINITY_DN113122_c0_g1_i1_1	60_58	alkaline phosphatase
TRINITY_DN524240_c0_g1_i1_1	60_8	nitrogen regulation protein NR(I)
TRINITY_DN234478_c0_g1_i1_1	62_12	septum formation initiator subfamily
TRINITY_DN329760_c0_g1_i1_1	62_13	phosphopyruvate hydratase
TRINITY_DN537573_c0_g1_i1_1	62_15	CTP synthase
TRINITY_DN516343_c0_g1_i1_1	634_1	carbamoyl phosphate synthase large subunit
TRINITY_DN211602_c0_g4_i1_1	65_17	ABC transporter substrate-binding protein
TRINITY_DN162247_c0_g1_i1_1	65_19	C4-dicarboxylate ABC transporter
TRINITY_DN215552_c1_g1_i1_1	65_22	phosphate ABC transporter permease subunit PstC
TRINITY_DN228878_c0_g1_i1_1	65_38	type IV pili twitching motility protein PilT
TRINITY_DN240799_c0_g1_i1_2	65_40	YggS family pyridoxal phosphate enzyme
TRINITY_DN439225_c0_g1_i1_1	67_19	fructose-1,6-bisphosphate aldolase
TRINITY_DN552143_c0_g1_i1_1	67_24	methionine adenosyltransferase
TRINITY_DN460265_c0_g1_i1_1	67_61	glutamine amidotransferase
TRINITY_DN4267_c0_g1_i1_1	70_20	flavin-binding monooxygenase AlmA
TRINITY_DN318388_c0_g1_i1_1	70_23	3'(2'),5'-bisphosphate nucleotidase CysQ
TRINITY_DN39899_c1_g1_i1_1	70_5	ABC transporter ATP-binding protein
TRINITY_DN492461_c0_g1_i1_1	70_7	isocitrate dehydrogenase
TRINITY_DN177727_c0_g1_i1_2	71_7	GTP-binding protein TypA
TRINITY_DN450863_c0_g1_i1_1	72_8	2-nitropropane dioxygenase
TRINITY_DN184100_c0_g1_i1_1	804_4	valine--tRNA ligase
TRINITY_DN552222_c0_g1_i1_1	81_16	hypothetical protein

TRINITY_DN186106_c0_g1_i1_2	81_23	hemolysin
TRINITY_DN158618_c0_g1_i1_1	85_49	signal recognition particle protein
TRINITY_DN206010_c0_g1_i1_1	85_53	50S ribosomal protein L19
TRINITY_DN237521_c0_g2_i4_1	86_39	transporter
TRINITY_DN218681_c0_g1_i1_1	90_38	3-dehydroquinate dehydratase
TRINITY_DN22045_c0_g1_i1_1	94_19	tRNA (N6-isopentenyl adenosine(37)-C2)-methylthiotransferase MiaB
TRINITY_DN198481_c0_g3_i1_2	94_3	leucine--tRNA ligase

Table S4. Transcripts of *Alcanivorax jadensis* C13 in the metatranscriptomes..

Transcriptome	Contig ID in Transcriptome	*A*. *jadensis* C13 gene ID	Annotation
DY37-CTD01	TRINITY_DN203_c0_g1_i2_2	184_6	50S ribosomal protein L2
	TRINITY_DN1338_c0_g1_i1_1	132_43	glutamyl-tRNA amidotransferase
DY37-CTD02	TRINITY_DN94_c0_g1_i1_1	255_33	biotin--acetyl-CoA-carboxylase ligase
	TRINITY_DN94_c0_g2_i1_1	17401_2	phosphonate ABC transporter permease
DY37-CTD03	TRINITY_DN509_c0_g1_i1_1	415_25	30S ribosomal protein S1
	TRINITY_DN77_c0_g1_i1_1	16560_4	phage tail tape measure protein
	TRINITY_DN0_c0_g1_i1_1	184_6	50S ribosomal protein L2
DY37-CTD04	-	-	-
DY37-CTD05	TRINITY_DN1288_c0_g1_i1_1	17401_2	phosphonate ABC transporter permease
	TRINITY_DN1656_c0_g1_i1_1	11_57	cytochrome B
DY37-CTD06	TRINITY_DN1201_c0_g1_i1_1	415_25	30S ribosomal protein S1
TS01-CTD07	TRINITY_DN312715_c0_g1_i1_1	1_11	monovalent cation/H+ antiporter subunit A
	TRINITY_DN556115_c0_g1_i1_1	1_13	monovalent cation/H+ antiporter subunit D
	TRINITY_DN460265_c0_g1_i1_1	1_34	glutathione peroxidase
	TRINITY_DN238473_c0_g5_i2_1	1_69	adenosylhomocysteinase
	TRINITY_DN552143_c0_g1_i1_1	1_70	methionine adenosyltransferase
	TRINITY_DN100973_c0_g1_i1_1	1_72	transketolase
	TRINITY_DN439225_c0_g1_i1_1	1_75	fructose-1,6-bisphosphate aldolase
	TRINITY_DN212486_c0_g3_i1_1	1_102	glycine dehydrogenase (aminomethyl-transferring), partial
	TRINITY_DN318388_c0_g1_i1_1	1_180	3'(2'),5'-bisphosphate nucleotidase CysQ
	TRINITY_DN4267_c0_g1_i1_1	1_183	flavin-binding monooxygenase AlmA
	TRINITY_DN492461_c0_g1_i1_1	1_196	isocitrate dehydrogenase
	TRINITY_DN39899_c1_g1_i1_1	1_198	ABC transporter ATP-binding protein
	TRINITY_DN71274_c0_g1_i1_1	1_230	DNA topoisomerase IV subunit B
	TRINITY_DN201910_c0_g1_i1_1	1_231	DNA topoisomerase IV subunit A

TRINITY_DN515931_c0_g1_i1_1	5_36	ribonuclease PH
TRINITY_DN310345_c0_g1_i1_1	5_40	dihydroxy-acid dehydratase
TRINITY_DN232554_c0_g2_i1_1	5_43	(p)ppGpp synthetase
TRINITY_DN183936_c0_g1_i1_1	5_76	peptide deformylase
TRINITY_DN240799_c0_g1_i1_2	5_103	YggS family pyridoxal phosphate enzyme
TRINITY_DN236436_c0_g10_i1_1	5_104	twitching motility protein PilT
TRINITY_DN228878_c0_g1_i1_1	5_105	type IV pili twitching motility protein PilT
TRINITY_DN215552_c1_g1_i1_1	5_121	phosphate ABC transporter permease subunit PstC
TRINITY_DN162247_c0_g1_i1_1	5_124	C4-dicarboxylate ABC transporter
TRINITY_DN211602_c0_g4_i1_1	5_126	ABC transporter substrate-binding protein
TRINITY_DN275304_c0_g1_i1_1	7_119	tRNA uridine-5-carboxymethylaminomethyl(34) synthesis enzyme MnmG
TRINITY_DN139945_c0_g1_i1_1	7_131	F0F1 ATP synthase subunit beta
TRINITY_DN195640_c0_g1_i1_1	7_133	UDP-N-acetylglucosamine diphosphorylase/glucosamine-1-phosphate N-acetyltransferase
TRINITY_DN464907_c0_g1_i1_1	7_134	glutamine--fructose-6-phosphate aminotransferase
TRINITY_DN195215_c0_g1_i1_1	7_61	histone deacetylase superfamily protein
TRINITY_DN193314_c0_g1_i1_1	7_87	hypothetical protein
TRINITY_DN325258_c0_g1_i1_1	7_9	hybrid sensor histidine kinase/response regulator
TRINITY_DN186736_c0_g1_i1_1	9_35	hypothetical protein
TRINITY_DN172336_c0_g1_i1_1	9_39	isovaleryl-CoA dehydrogenase
TRINITY_DN111826_c0_g1_i1_1	9_81	NADP-dependent isocitrate dehydrogenase
TRINITY_DN509335_c0_g1_i1_1	9_84	ATP-dependent Clp protease ATP-binding subunit ClpA
TRINITY_DN186359_c0_g1_i1_1	9_91	recombination factor protein RarA
TRINITY_DN230719_c0_g1_i1_1	9_93	serine--tRNA ligase
TRINITY_DN106482_c0_g2_i1_2	9_101	arsenic-transporting ATPase
TRINITY_DN354141_c0_g1_i1_1	9_111	chemotaxis protein CheY
TRINITY_DN219286_c0_g1_i1_1	9_126	methionine synthase
TRINITY_DN182079_c0_g1_i1_1	10_3	anhydro-N-acetylmuramic acid kinase
TRINITY_DN436498_c0_g1_i1_1	10_15	glutamate-1-semialdehyde-2,1-aminomutase

TRINITY_DN191147_c1_g1_i1_1	10_35	acetate--CoA ligase
TRINITY_DN516343_c0_g1_i1_1	10_52	carbamoyl phosphate synthase large subunit
TRINITY_DN273179_c0_g1_i1_1	10_65	molecular chaperone DnaJ
TRINITY_DN364200_c0_g1_i1_1	10_99	elongation factor P
TRINITY_DN519803_c0_g1_i1_1	11_23	type II secretion system protein F
TRINITY_DN330674_c0_g1_i1_1	11_37	UDP-N-acetylmuramate--L-alanine ligase
TRINITY_DN184011_c0_g1_i1_2	11_85	ABC transporter ATP-binding protein
TRINITY_DN168952_c0_g1_i1_1	11_120	ribose-phosphate pyrophosphokinase
TRINITY_DN153548_c0_g1_i1_1	11_121	50S ribosomal protein L25/general stress protein Ctc
TRINITY_DN173611_c0_g1_i1_1	12_7	cell division ATP-binding protein FtsE
TRINITY_DN450863_c0_g1_i1_1	12_70	2-nitropropane dioxygenase
TRINITY_DN177727_c0_g1_i1_2	27_48	GTP-binding protein TypA
TRINITY_DN524240_c0_g1_i1_1	27_58	nitrogen regulation protein NR(I)
TRINITY_DN257439_c0_g1_i1_1	27_77	arginine--tRNA ligase
TRINITY_DN106945_c0_g1_i1_1	27_78	primosomal protein N'
TRINITY_DN198893_c0_g3_i1_1	27_80	malate dehydrogenase
TRINITY_DN389716_c0_g1_i1_1	27_81	peptidase
TRINITY_DN358538_c0_g1_i1_1	34_15	NADH:ubiquinone reductase (Na(+)-transporting) subunit D
TRINITY_DN552222_c0_g1_i1_1	34_38	hypothetical protein
TRINITY_DN186106_c0_g1_i1_2	34_45	cysteine synthase A
TRINITY_DN70368_c0_g1_i1_1	41_7	tryptophan synthase subunit beta
TRINITY_DN329540_c0_g1_i1_1	41_44	succinate--CoA ligase subunit alpha
TRINITY_DN518378_c0_g1_i1_1	41_53	citrate (Si)-synthase
TRINITY_DN231767_c0_g5_i1_1	43_10	hypothetical protein
TRINITY_DN339105_c0_g1_i1_1	43_62	NAD-glutamate dehydrogenase
TRINITY_DN438986_c0_g1_i1_1	43_68	class II aldolase
TRINITY_DN400121_c0_g1_i1_1	54_4	acyl-CoA dehydrogenase
TRINITY_DN206010_c0_g1_i1_1	55_25	50S ribosomal protein L19
TRINITY_DN158618_c0_g1_i1_1	55_29	signal recognition particle protein
TRINITY_DN269749_c0_g1_i1_1	59_26	copper-translocating P-type ATPase

TRINITY_DN197518_c0_g1_i1_1	59_53	argininosuccinate lyase
TRINITY_DN486274_c0_g1_i1_1	59_67	dihydroxy-acid dehydratase
TRINITY_DN238251_c1_g4_i1_1	63_34	RNA helicase
TRINITY_DN226172_c0_g1_i1_1	63_37	ATP-dependent DNA helicase RecQ
TRINITY_DN24748_c0_g1_i1_1	63_43	proline--tRNA ligase
TRINITY_DN126417_c0_g1_i1_1	77_1	aldehyde dehydrogenase
TRINITY_DN185810_c1_g1_i1_1	80_2	glutamate synthase large subunit
TRINITY_DN113122_c0_g1_i1_1	80_20	alkaline phosphatase
TRINITY_DN397681_c0_g1_i1_1	80_40	adenylosuccinate synthase
TRINITY_DN233885_c0_g1_i1_2	80_50	30S ribosomal protein S6
TRINITY_DN225468_c0_g1_i1_1	87_58	alkane 1-monooxygenase
TRINITY_DN235328_c0_g1_i1_1	87_8	AAA family ATPase
TRINITY_DN234478_c0_g1_i1_1	93_9	septum formation initiator subfamily
TRINITY_DN329760_c0_g1_i1_1	93_10	phosphopyruvate hydratase
TRINITY_DN537573_c0_g1_i1_1	93_12	CTP synthase
TRINITY_DN232442_c0_g4_i1_1	100_9	ATP-dependent chaperone ClpB
TRINITY_DN149908_c1_g1_i1_1	100_13	NAD+ synthase
TRINITY_DN323425_c0_g1_i1_2	100_25	4-hydroxy-3-methylbut-2-enyl diphosphate reductase
TRINITY_DN224675_c0_g2_i1_1	100_28	isoleucine--tRNA ligase
TRINITY_DN168467_c0_g2_i1_1	100_42	short chain dehydrogenase
TRINITY_DN218681_c0_g1_i1_1	114_19	3-dehydroquinate dehydratase
TRINITY_DN215163_c0_g3_i1_1	118_10	glyceraldehyde 3-phosphate dehydrogenase
TRINITY_DN161529_c0_g1_i1_1	118_27	alanine--tRNA ligase
TRINITY_DN167802_c0_g2_i1_1	130_11	peptide chain release factor 3
TRINITY_DN571068_c0_g1_i1_1	132_41	tRNA (adenosine(37)-N6)-threonylcarbamoyltransferase complex transferase subunit TsaD
TRINITY_DN193839_c0_g3_i1_1	132_45	RNA polymerase sigma factor RpoD
TRINITY_DN537943_c0_g1_i1_1	135_20	cytochrome c oxidase subunit I
TRINITY_DN404282_c0_g1_i1_1	135_25	RNA polymerase-binding ATPase
TRINITY_DN22045_c0_g1_i1_1	148_26	tRNA (N6-isopentenyl adenosine(37)-C2)-methylthiotransferase MiaB

TRINITY_DN198481_c0_g3_i1_2	148_42	leucine--tRNA ligase
TRINITY_DN530913_c0_g1_i1_1	162_33	acyl-CoA dehydrogenase
TRINITY_DN543762_c1_g1_i1_1	184_1	elongation factor Tu
TRINITY_DN151558_c0_g2_i1_1	184_10	50S ribosomal protein L16
TRINITY_DN469651_c0_g1_i1_1	184_23	preprotein translocase subunit SecY
TRINITY_DN224335_c0_g3_i1_1	192_24	5-methyltetrahydropteroyltriglutamate--homocysteine S-methyltransferase
TRINITY_DN169630_c0_g1_i1_1	195_37	citrate synthase/methylcitrate synthase
TRINITY_DN324782_c0_g1_i1_1	212_3	ATP-dependent Clp protease ATP-binding subunit ClpX
TRINITY_DN175259_c0_g1_i1_1	212_4	endopeptidase La
TRINITY_DN239794_c0_g1_i1_1	212_8	ABC transporter ATP-binding protein
TRINITY_DN453910_c0_g1_i1_1	212_10	microcin ABC transporter permease
TRINITY_DN239306_c0_g1_i1_1	212_30	ABC1 family protein, ubiquinone biosynthesis protein
TRINITY_DN477983_c0_g1_i1_1	230_1	single-stranded-DNA-specific exonuclease RecJ
TRINITY_DN238543_c1_g1_i1_1	230_17	elongation factor 4
TRINITY_DN272025_c0_g1_i1_1	230_25	cysteine synthase B
TRINITY_DN175844_c0_g1_i1_1	255_33	biotin--acetyl-CoA-carboxylase ligase
TRINITY_DN194388_c0_g1_i1_1	259_12	aconitate hydratase
TRINITY_DN93534_c0_g1_i1_1	292_10	electron transfer flavoprotein-ubiquinone oxidoreductase
TRINITY_DN378667_c0_g1_i1_1	308_22	ABC-F family ATPase
TRINITY_DN486361_c0_g1_i1_1	333_20	acetyl-CoA C-acyltransferase FadA
TRINITY_DN542343_c0_g1_i1_1	333_3	ATP-dependent RNA helicase DbpA
TRINITY_DN237521_c0_g2_i4_1	342_17	transporter
TRINITY_DN520113_c0_g1_i1_1	414_25	acyl-CoA dehydrogenase
TRINITY_DN285341_c0_g1_i1_1	415_25	30S ribosomal protein S1
TRINITY_DN400860_c0_g1_i1_1	484_6	acetyl-coenzyme A synthetase
TRINITY_DN448109_c0_g1_i1_1	489_12	Fe-S cluster assembly protein SufB
TRINITY_DN403199_c0_g1_i1_1	527_9	acyl-CoA dehydrogenase
TRINITY_DN530645_c0_g1_i1_1	945_7	hypothetical protein
TRINITY_DN235232_c0_g1_i2_1	1340_10	radical SAM/Cys-rich domain protein

TRINITY_DN143182_c0_g1_i1_1	1340_5	transducer for aerotaxis sensory component ; methyl accepting chemotaxis component
TRINITY_DN301966_c0_g1_i1_1	1400_8	DNA gyrase subunit A
TRINITY_DN519677_c0_g1_i1_1	1484_8	cardiolipin synthase
TRINITY_DN220749_c1_g1_i1_1	1842_1	protoheme IX farnesyltransferase
TRINITY_DN368463_c0_g1_i1_1	1842_2	peroxiredoxin
TRINITY_DN400243_c0_g1_i1_1	1842_4	cytochrome-c oxidase
TRINITY_DN153923_c0_g1_i1_1	1842_5	cytochrome c oxidase subunit I
TRINITY_DN567082_c0_g1_i1_1	1842_7	bb3-type cytochrome oxidase subunit IV
TRINITY_DN410172_c0_g1_i1_1	1842_9	cytochrome c oxidase subunit II
TRINITY_DN363935_c0_g1_i1_1	2003_5	methionine synthase
TRINITY_DN213713_c0_g1_i1_1	2003_6	ergothioneine biosynthesis protein EgtB
TRINITY_DN172174_c0_g1_i1_1	2003_7	glycosyl transferase
TRINITY_DN471471_c0_g1_i1_1	2003_8	selenophosphate synthase
TRINITY_DN229440_c1_g1_i1_1	2003_9	rhodanese-like domain-containing protein
TRINITY_DN524468_c0_g1_i1_2	2051_2	glutamate-5-semialdehyde dehydrogenase
TRINITY_DN308940_c0_g1_i1_1	3307_2	3-dehydroquinate synthase
TRINITY_DN337156_c0_g1_i1_1	3307_3	membrane protein
TRINITY_DN387954_c0_g1_i1_1	3307_9	molecular chaperone DnaJ
TRINITY_DN349364_c0_g1_i1_1	3417_1	lactate dehydrogenase, partial
TRINITY_DN162997_c0_g1_i1_2	3417_2	serine--glyoxylate aminotransferase
TRINITY_DN219532_c1_g1_i1_1	3417_3	enoyl-CoA hydratase
TRINITY_DN165484_c0_g1_i1_1	3417_4	CoA-transferase
TRINITY_DN519453_c0_g1_i1_1	3417_5	hypothetical protein
TRINITY_DN275727_c0_g1_i1_1	3417_7	hypothetical protein
TRINITY_DN585096_c0_g1_i1_1	3700_4	valine--tRNA ligase
TRINITY_DN470077_c0_g1_i1_1	4067_2	L-selenocysteinyl-tRNA(Sec) synthase
TRINITY_DN337256_c0_g1_i1_2	4067_4	RNA polymerase subunit sigma-24
TRINITY_DN396728_c0_g1_i1_1	4067_5	hypothetical protein AMJ77_02895
TRINITY_DN190088_c0_g1_i1_1	4067_6	hypothetical protein AMJ37_01390

TRINITY_DN63070_c0_g1_i1_1	4580_1	glycolate dehydrogenase, subunit GlcD
TRINITY_DN306204_c0_g1_i1_1	4960_1	transposase
TRINITY_DN179340_c0_g3_i1_1	4960_2	IS110 family transposase
TRINITY_DN239510_c4_g6_i1_1	4960_3	IS110 family transposase
TRINITY_DN574987_c0_g1_i1_2	5069_1	branched-chain amino acid ABC transporter permease
TRINITY_DN574987_c0_g1_i1_1	5069_2	branched-chain amino acid ABC transporter permease
TRINITY_DN180873_c0_g1_i1_1	5069_3	ABC transporter ATP-binding protein
TRINITY_DN236695_c1_g1_i1_1	5069_4	ABC transporter ATP-binding protein
TRINITY_DN324115_c0_g1_i1_1	5069_5	hypothetical protein
TRINITY_DN170414_c0_g1_i1_1	5286_2	formate dehydrogenase subunit alpha
TRINITY_DN210139_c0_g1_i1_1	5286_3	NADH-quinone oxidoreductase subunit F
TRINITY_DN429577_c0_g1_i1_1	6267_2	hypothetical protein
TRINITY_DN319777_c0_g1_i1_1	6267_5	ferrous iron transporter
TRINITY_DN165326_c0_g1_i1_1	6390_1	acetyl-CoA acetyltransferase
TRINITY_DN500758_c0_g1_i1_1	6390_2	long-chain-fatty-acid--CoA ligase
TRINITY_DN248367_c0_g1_i1_1	6390_3	hypothetical protein
TRINITY_DN239533_c3_g7_i1_1	6663_2	ABC transporter substrate-binding protein
TRINITY_DN181430_c0_g1_i1_1	6663_3	TRAP ABC transporter permease
TRINITY_DN375100_c0_g1_i1_1	6663_4	C4-dicarboxylate ABC transporter
TRINITY_DN84565_c0_g1_i1_1	6873_1	osmotically inducible protein C
TRINITY_DN376420_c0_g1_i1_1	6873_2	hypothetical protein
TRINITY_DN218835_c0_g1_i1_1	6873_3	class I poly(R)-hydroxyalkanoic acid synthase
TRINITY_DN204081_c0_g1_i1_1	9306_1	transcriptional repressor
TRINITY_DN106880_c0_g1_i1_1	9306_3	ABC transporter
TRINITY_DN236441_c0_g1_i1_1	11359_2	ATPase AAA
TRINITY_DN286020_c0_g1_i1_1	12299_1	NADH:ubiquinone reductase (Na(+)-transporting) subunit E
TRINITY_DN127866_c0_g1_i1_1	13010_2	Ammonia channel precursor
TRINITY_DN184059_c0_g1_i1_1	13869_1	polyribonucleotide nucleotidyltransferase
TRINITY_DN432459_c0_g1_i1_1	13869_2	3-hydroxyacyl-
TRINITY_DN566780_c0_g1_i1_1	15908_1	diguanylate cyclase

TRINITY_DN223444_c0_g1_i1_1	15908_2	hypothetical protein AYK26_05005
TRINITY_DN236486_c0_g1_i1_1	15908_3	putative sugar (and other) transporter
TRINITY_DN236486_c0_g4_i1_2	15908_4	putative sugar (and other) transporter
TRINITY_DN349847_c0_g1_i1_1	16560_4	phage tail tape measure protein
TRINITY_DN208647_c0_g1_i1_1	16665_1	protease TldD
TRINITY_DN181331_c0_g1_i1_1	17401_2	phosphonate ABC transporter permease
TRINITY_DN260269_c0_g1_i1_1	17948_1	formate dehydrogenase
TRINITY_DN55565_c0_g1_i1_1	17948_2	hypothetical protein
TRINITY_DN240436_c0_g7_i1_1	20396_1	imidazole glycerol phosphate synthase subunit HisH
TRINITY_DN170144_c0_g1_i1_2	20396_2	imidazole glycerol phosphate synthase subunit HisH
TRINITY_DN69561_c0_g1_i1_2	20396_3	hypothetical protein
TRINITY_DN447625_c0_g1_i1_1	20396_4	imidazoleglycerol-phosphate dehydratase
TRINITY_DN61287_c0_g2_i1_1	23903_2	hypothetical protein
TRINITY_DN222308_c0_g1_i1_1	23903_3	short-chain dehydrogenase

Figure S1. Microbial community structure in water samples based on sequencing of 16S rRNA gene amplicons.

Alcanivorax was marked by red frame. The samples are referred to Table S1.

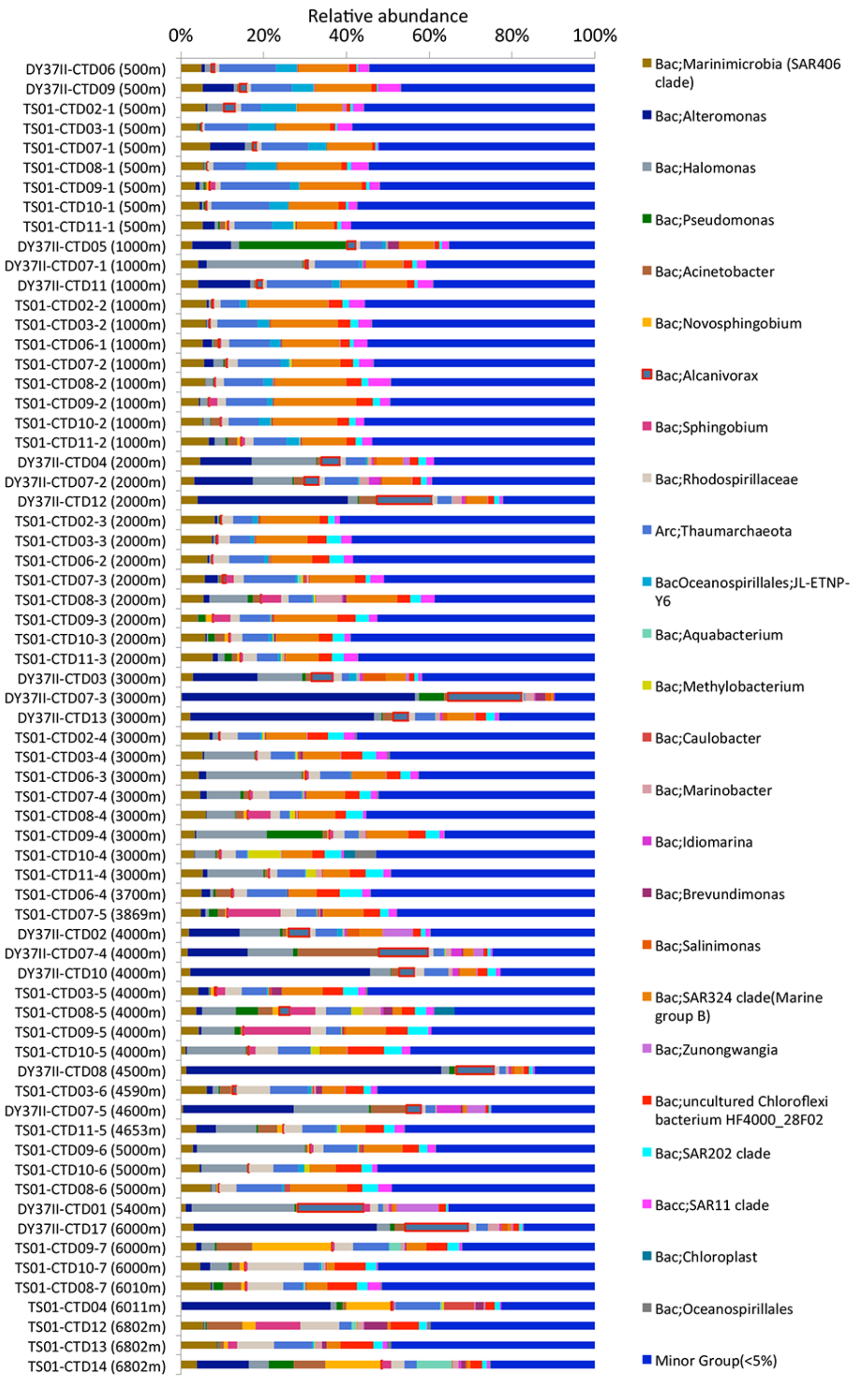

Figure S2. Microbial community structure in the samples collected by Jiaolong manned submersible. The diving sites of the Jiaolong submersible were displayed in Figure 1. Four bottom water samples (sample IDs ending with W) and three surface sediments (sample IDs ending with S) were used for the analysis. The microbial community structures in the samples were revealed by sequencing of 16S rRNA gene amplicons.

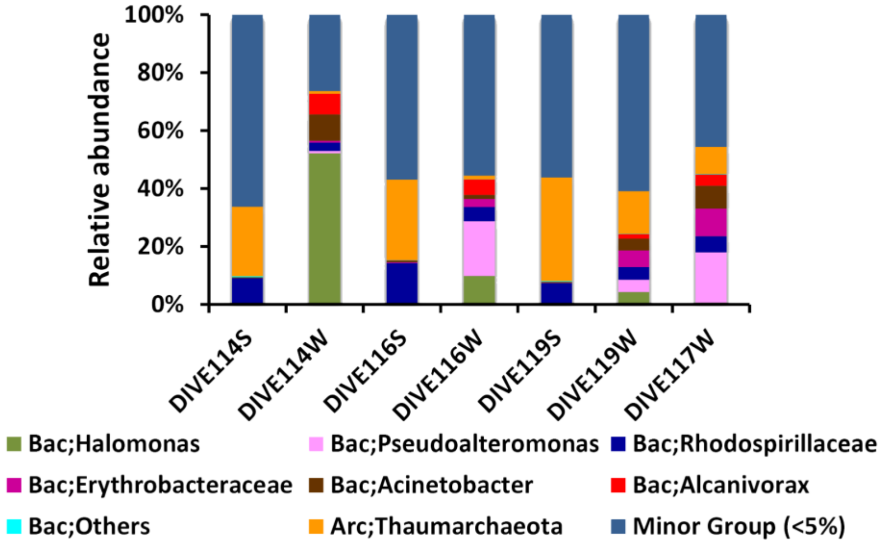

Figure S3. Genome binning.

Based on the G+C content and coverage by reads, the contigs with high coverage levels (A for DY37II-CTD13 & C for DY37II- CTD17) were binned for examination of the tetranucleotide frequency consistency in the CA analysis (B for DY37II- CTD13 & D for DY37II- CTD17).

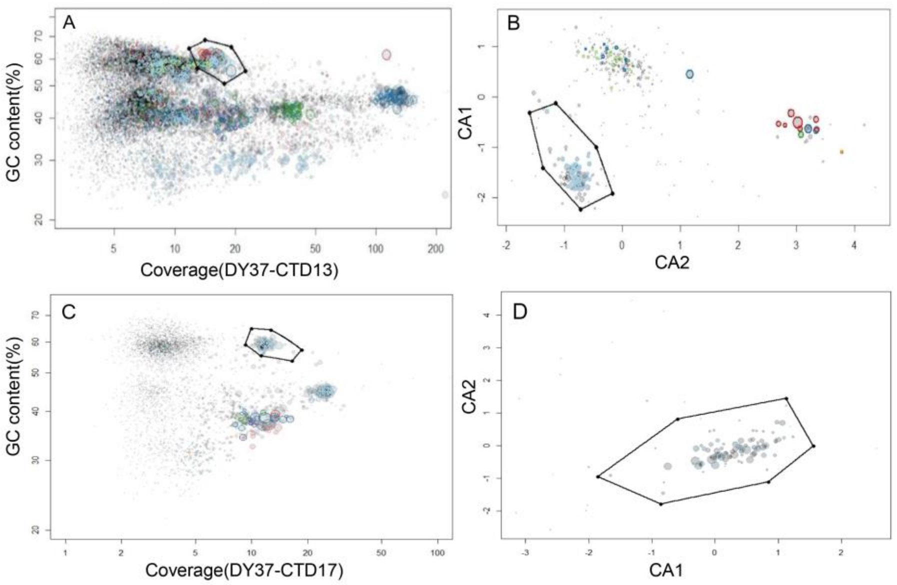

Figure S4. Number of KEGG genes in *Alcanivorax* genomes.

Five genomes for *Alcanivorax jadensis* C13, *Alcanivorax jadensis* C17, *Alcanivorax jadensis* T9, *Alcanivorax borkumensis* SK2, and *Alcanivorax dieselolei* B5, respectively, were used for the comparison.

KEGG	*A. jadsensis* C13	*A. jadsensis* C17	*A. borkumensis* SK2	*A. jadsensis* T9	*A. dieselolei* B5	
K01623	1	1	0	0	0	ALDO; fructose-bisphosphate aldolase, class I
K01810	0	0	1	1	1	GPI,_pgi;_glucose-6-phosphate_isomerase
K00330	1	0	0	0	0	nuoA; NADH-quinone oxidoreductase subunit A
K00331	1	0	0	0	0	nuoB; NADH-quinone oxidoreductase subunit B
K00335	1	0	0	0	0	nuoF; NADH-quinone oxidoreductase subunit F
K00340	1	0	0	0	0	nuoK; NADH-quinone oxidoreductase subunit K
K00342	1	0	0	0	0	nuoM; NADH-quinone oxidoreductase subunit M
K00343	1	0	0	0	0	nuoN; NADH-quinone oxidoreductase subunit N
K00356	2	2	2	2	2	E1.6.99.3; NADH dehydrogenase
K03885	1	1	1	1	1	ndh; NADH dehydrogenase
K15576	3	2	2	2	2	nrtA, nasF, cynA; nitrate/nitrite transport system subs
K15577	2	1	1	1	1	nrtB, nasE, cynB; nitrate/nitrite transport system perm
K15578	2	1	1	1	1	nrtC, nasD; nitrate/nitrite transport system ATP-bindi
K00370	0	0	1	0	1	narG, narZ, nxrA; nitrate reductase / nitrite oxidoredu
K00371	0	0	1	0	1	narH, narY, nxrB; nitrate reductase / nitrite oxidoredu
K00374	0	0	1	0	1	narI, narV; nitrate reductase gamma subunit
K00372	2	1	1	1	1	nasA; assimilatory nitrate reductase catalytic subunit
K00362	5	2	3	2	2	nirB; nitrite reductase (NADH) large subunit
K00363	4	2	2	2	1	nirD; nitrite reductase (NADH) small subunit
K00368	0	0	1	1	1	nirK; nitrite reductase (NO-forming)
K04561	0	0	0	0	1	norB; nitric oxide reductase subunit B
K00376	0	0	0	0	1	nosZ; nitrous-oxide reductase
K00459	5	5	2	5	6	ncd2, npd; nitronate monooxygenase
K00249	9	12	11	12	25	acd; acyl-CoA dehydrogenase
K01692	7	6	5	6	16	paaF, echA; enoyl-CoA hydratase
K01728	4	4	0	4	0	pel; pectate lyase

Figure S5. Microbial communities in samples collected by two cruises.

Taxonomic assignment was conducted using the RDP classifier by referring to the SILVA database. Only the microbes accounted for >1% of the whole communities were shown.

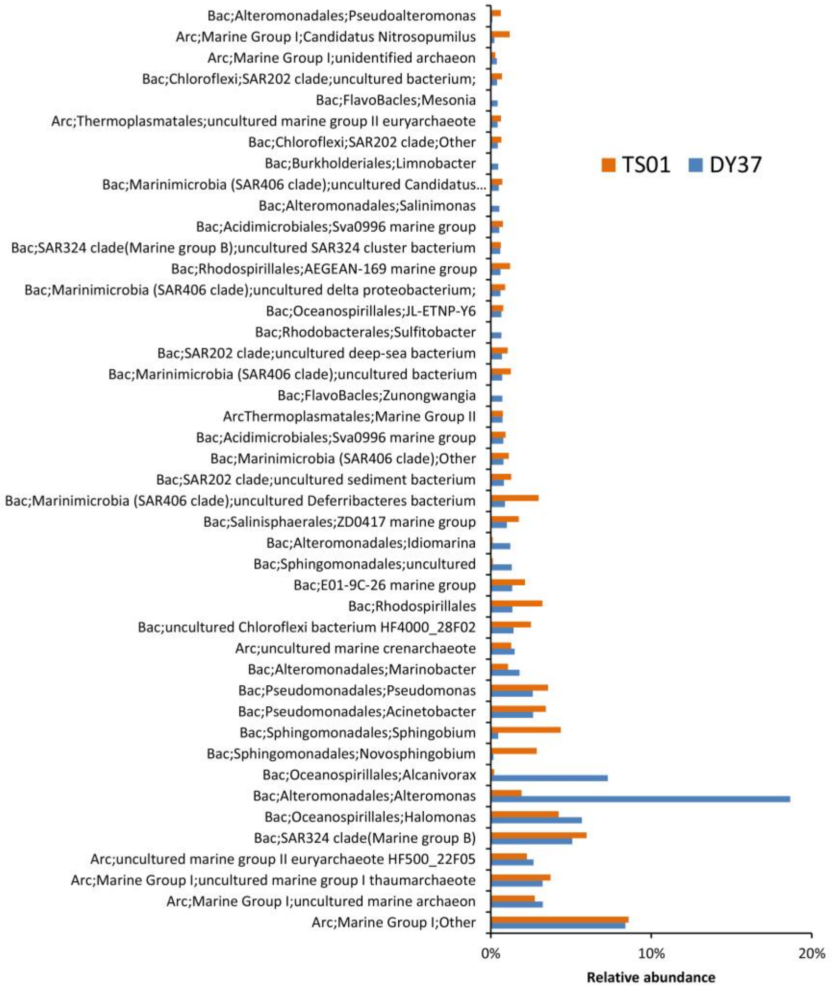

Figure S6. A pockmark photographed by Jiaolong manned submersible.

The pockmark was located in northern slope of the Mariana Trench (142.202 E, 10.885 N).

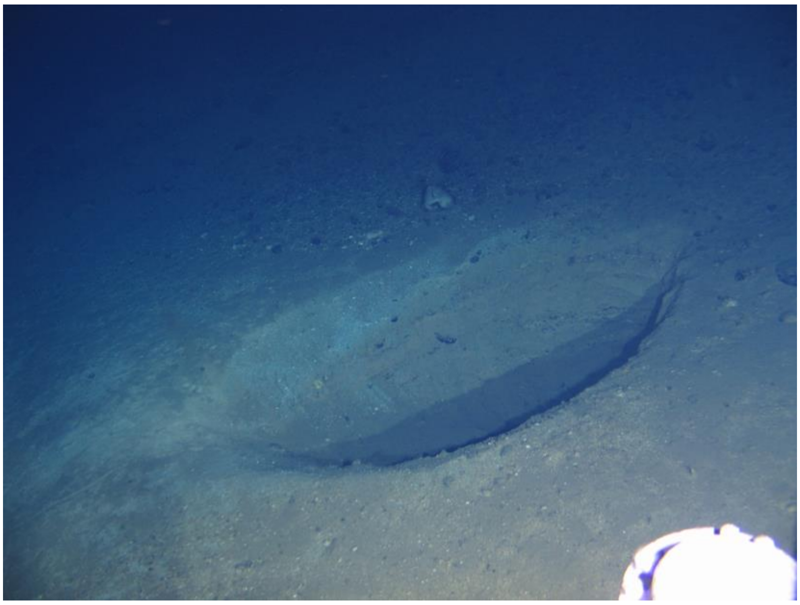

Figure S7. The GCMS result of water samples.

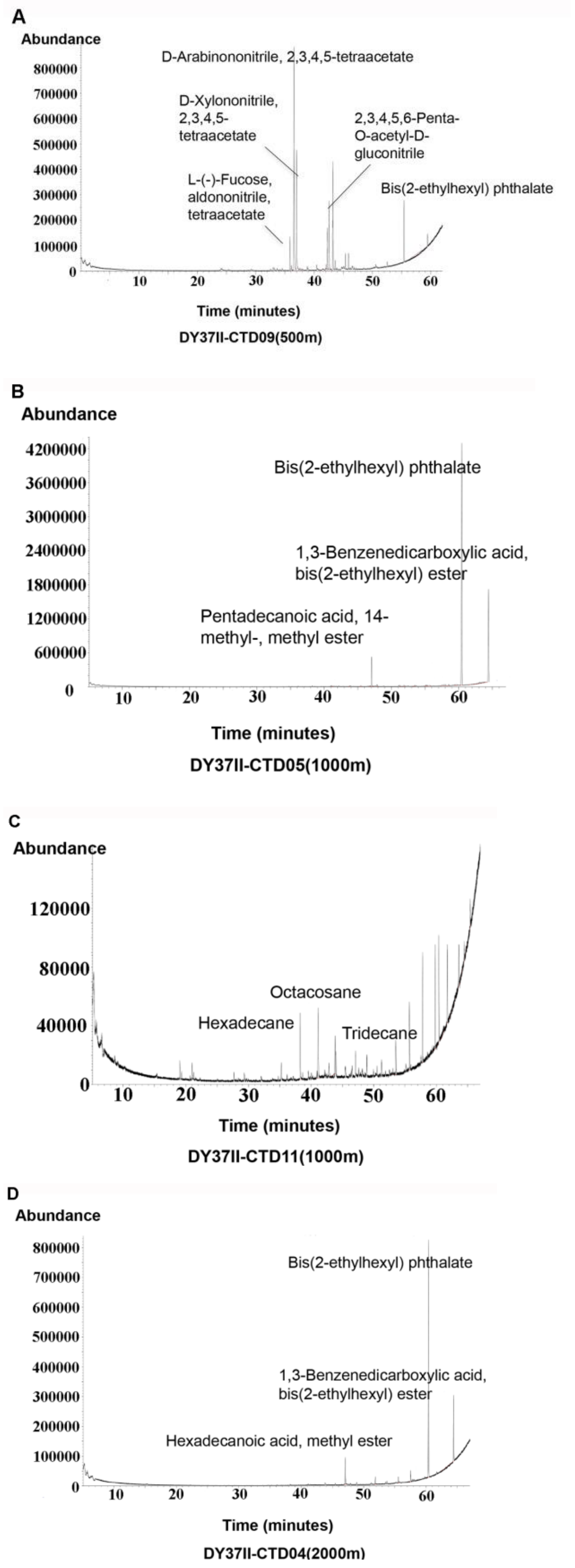

E

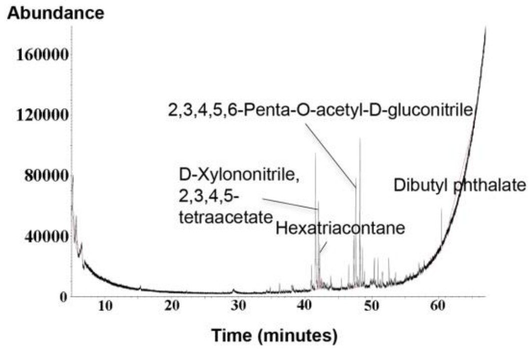

DY37II-CTD12(2000m)

F

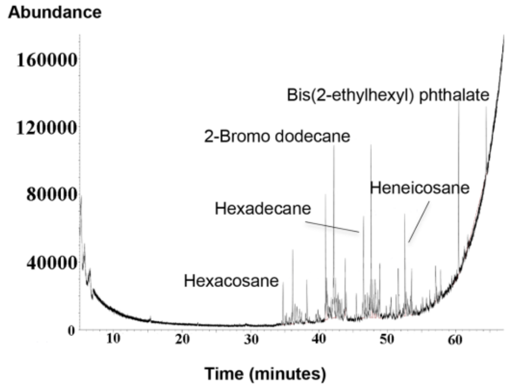

DY37II-CTD13(3000m)

G

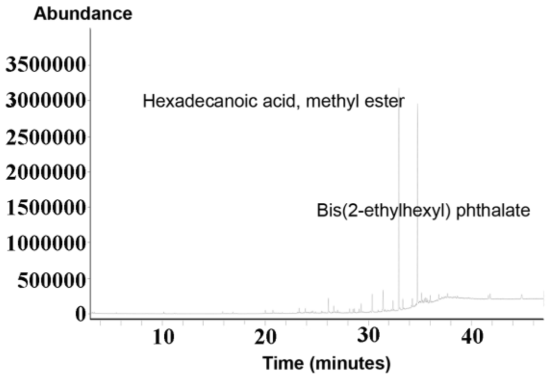

TS01-CTS07-5(3869m)

H

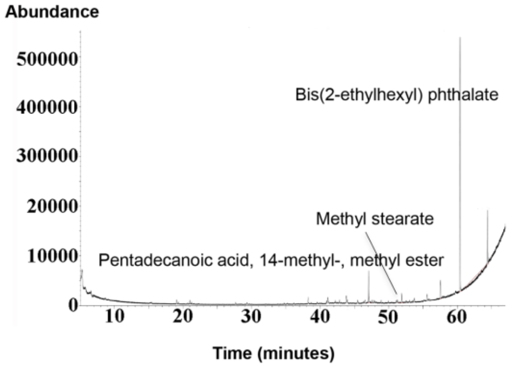

DY37II-CTD02(4000m)

I

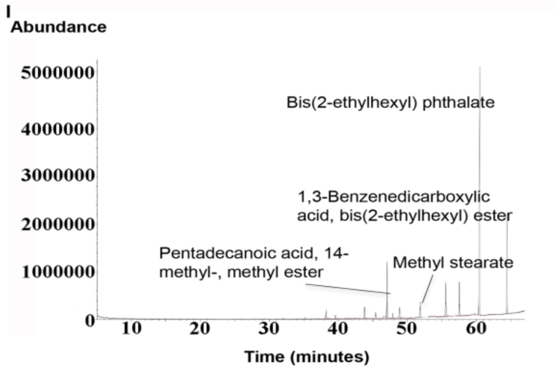

DY37II-CTD10(4000m)

J

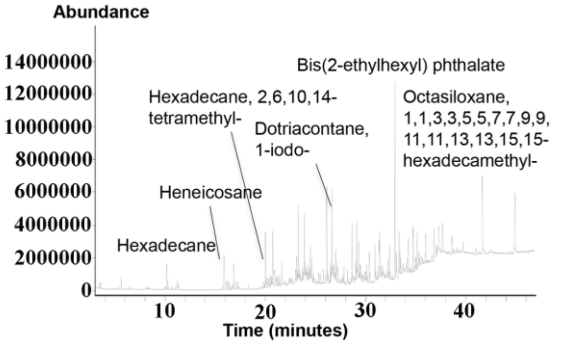

DIVE114(water,5467m)

K

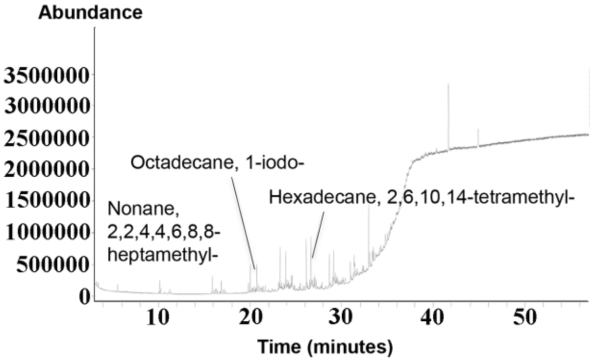

DIVE119(water,6000m)

L

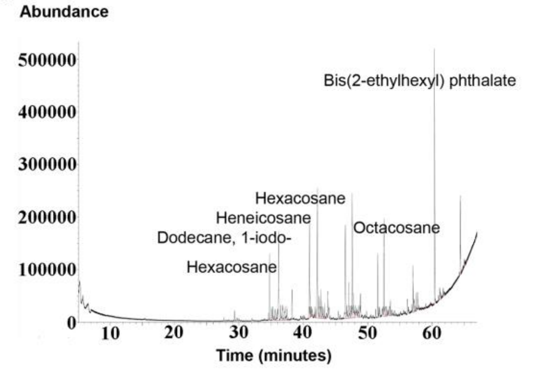

DY37II-CTD17(6000m)

M

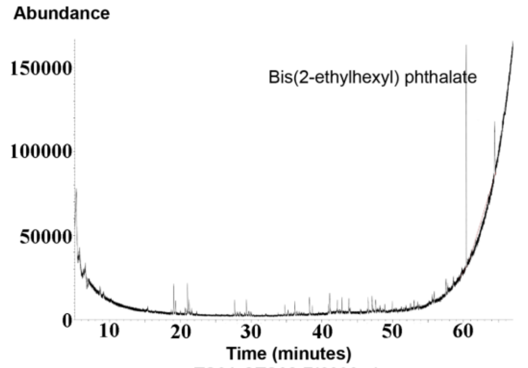

TS01-CTS08-7(6000m)

N

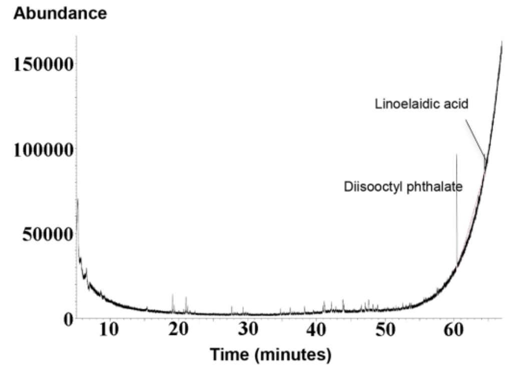

TS01-CTS09-7(6000m)

O

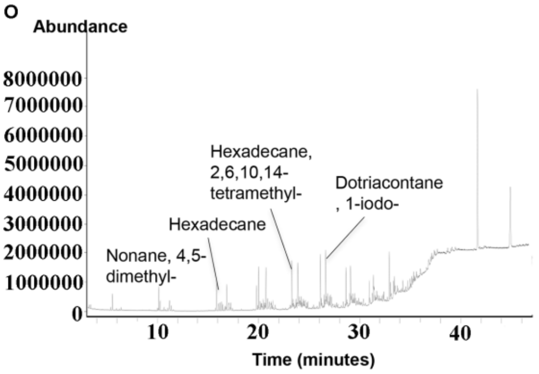

TS01-CTS04(6000m)

P

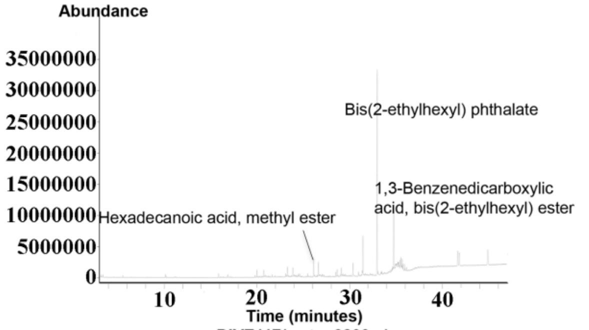

DIVE117(water,6200m)
